# Optimization of a Two-Species Microbial Consortium for Improved Mcl-PHA Production From Glucose–Xylose Mixtures

**DOI:** 10.3389/fbioe.2021.794331

**Published:** 2022-01-10

**Authors:** Yinzhuang Zhu, Mingmei Ai, Xiaoqiang Jia

**Affiliations:** ^1^ Department of Biochemical Engineering, School of Chemical Engineering and Technology, Tianjin University, Tianjin, China; ^2^ Frontier Science Center for Synthetic Biology and Key Laboratory of Systems Bioengineering (MOE), School of Chemical Engineering and Technology, Tianjin University, Tianjin, China; ^3^ Collaborative Innovation Center of Chemical Science and Engineering, Tianjin, China

**Keywords:** MCL-PHA, artificial microbial consortium, engineered *Escherichia coli*, engineered *Pseudomonas putida*, xylose

## Abstract

Polyhydroxyalkanoates (PHAs) have attracted much attention as a good substitute for petroleum-based plastics, especially mcl-PHA due to their superior physical and mechanical properties with broader applications. Artificial microbial consortia can solve the problems of low metabolic capacity of single engineered strains and low conversion efficiency of natural consortia while expanding the scope of substrate utilization. Therefore, the use of artificial microbial consortia is considered a promising method for the production of mcl-PHA. In this work, we designed and constructed a microbial consortium composed of engineered *Escherichia coli* MG1655 and *Pseudomonas putida* KT2440 based on the “nutrition supply–detoxification” concept, which improved mcl-PHA production from glucose-xylose mixtures. An engineered *E. coli* that preferentially uses xylose was engineered with an enhanced ability to secrete acetic acid and free fatty acids (FFAs), producing 6.44 g/L acetic acid and 2.51 g/L FFAs with 20 g/L xylose as substrate. The mcl-PHA producing strain of *P. putida* in the microbial consortium has been engineered to enhance its ability to convert acetic acid and FFAs into mcl-PHA, producing 0.75 g/L mcl-PHA with mixed substrates consisting of glucose, acetic acid, and octanoate, while also reducing the growth inhibition of *E. coli* by acetic acid. The further developed artificial microbial consortium finally produced 1.32 g/L of mcl-PHA from 20 g/L of a glucose–xylose mixture (1:1) after substrate competition control and process optimization. The substrate utilization and product synthesis functions were successfully divided into the two strains in the constructed artificial microbial consortium, and a mutually beneficial symbiosis of “nutrition supply–detoxification” with a relatively high mcl-PHA titer was achieved, enabling the efficient accumulation of mcl-PHA. The consortium developed in this study is a potential platform for mcl-PHA production from lignocellulosic biomass.

## Introduction

Due to environmental problems such as energy waste and “white pollution” caused by petroleum-based plastics, the search for biodegradable alternatives to petroleum-based plastics has received increasing attention (Muneer et al., 2020). Polyhydroxyalkanoates (PHAs) are among the most well-studied biodegradable materials. Their polymer properties are similar to petroleum-based plastics, including low crystallinity, high tensile strength, high elongation at break, and low glass transition temperature (Chen and Patel, 2012), while also offering good biodegradability and biocompatibility, which makes them an excellent substitute for petroleum-based plastics (Raza et al., 2018). PHA is a polyester produced by microorganisms as energy storage particles in cells under nutritionally limited conditions (Lee, 1996), first discovered in *Bacillus megateriu*m in 1926 (Lemoigne, 1926). All types of PHA have similar structures but offer diversity in the monomer carbon chain length and side-chain groups, resulting in different material properties of various PHAs. Based on the repeat unit composition, more than 150 different PHA monomers have been identified so far, derived from hundreds of microorganisms (Agnew and Pfleger, 2013). There are many ways to classify PHA. According to the number of carbon atoms in the constituent monomers, PHA can be divided into short-chain-length polyhydroxyalkanoate (scl-PHA) composed of monomers with three to five carbon atoms, medium-chain-length polyhydroxyalkanoate (mcl-PHA) composed of monomers with 6–14 carbon atoms, and long-chain-length polyhydroxyalkanoate (lcl-PHA) composed of monomers with more than 15 carbon atoms (Singh and Mallick, 2009; Li et al., 2021). PHAs can also be classified according to the types of monomers, with homopolymers contain only one monomer, and copolymers containing more than one monomer. In terms of the physical properties, most scl-PHA has high crystallinity, brittleness, and hardness (Koning, 1995), except for individual monomers such as 4HB and 3HV, and et al. (Cavalheiro et al., 2013); while mcl-PHA is a thermoplastic, with low crystallinity, T_m_ values between 40 to 60°C, T_g_ values between −50 and −25°C, low tensile strength and high elongation at break (Kessler and Witholt, 1998).

Mcl-PHA has more diverse structures, as well as more flexible physical and mechanical properties, which can meet the needs of a broader range of engineering applications and realize material function customization (Chen and Wang, 2013). Previous studies found that mcl-PHA has better biocompatibility and biodegradability (Zhao et al., 2003a; Misra et al., 2006). In biological tissue engineering, PHA copolymers containing medium-chain length monomers exhibited higher biocompatibility than PHB (Liang et al., 2008). In medical applications, the impact of degradation products produced from PHA materials in cells is crucial. Several studies have proven that the biodegradability of PHA is affected by their physicochemical properties such as functional groups, affinity, hydrophobicity, structural order, and crystallinity (Zhao et al., 2002; Zhao et al., 2003b). Generally, the degradation ability of polymers decreases with the increase of crystallinity. Therefore, mcl-PHA with lower crystallinity and Tm has good biodegradability. In general, mcl-PHA has broader application prospects due to its better physical and mechanical properties, together with its environmental friendliness (Lee, 2006).

With the development of metabolic engineering and synthetic biology, natural (such as *Pseudomon*as) or non-natural (such as *E. coli*) PHA-producing strains can be engineered starting from the gene regulatory networks by regulating metabolic pathways, introducing the PHA synthesis pathway (Klinke et al., 1999; Zhuang et al., 2014), designing and synthesizing artificial cells, and building a promising metabolic platform for improved mcl-PHA accumulation (Borrero-de Acuna et al., 2014). Perhaps there are two main pathways to synthesize mcl-PHA in microorganisms, including the fatty acid β-oxidation pathway that metabolizes “related” carbon sources such as fatty acids and the fatty acid *de novo* synthesis pathway that uses “unrelated” carbon sources such as sugars (Tortajada et al., 2013). Huijberts et al. used the isotope labeling method to prove that when mcl-PHA was synthesized in *P. putida* KT2442, the fatty acid *de novo* synthesis pathway and fatty acid β-oxidation pathway were active at the same time but worked independently (Huijberts et al., 1994). At present, the related research on the production of mcl-PHA by single strains through the regulation of the above-mentioned metabolic pathways have been fully demonstrated. However, the limitation of a single engineered strain’s metabolic capacity results in a limited range of available substrates, high production costs, and low accumulation of mcl-PHA, which further hinders its large-scale production, popularization, and application. In recent years, microbial consortia that can complete the arduous task using compartmentalized metabolic pathways in two or more strains have received more attention. Using waste-activated sludge (WAS) to combine the PHA production process with the sewage treatment process can reduce costs (Liu et al., 2013; Valentino et al., 2015; Jayakrishnan et al., 2021). However, the natural diverse microbial consortium has a longer domestication cycle and is poorly controllable, leading to disadvantages compared with a single engineered strain (Morgan-Sagastume et al., 2015). Compared with a single engineered strain and natural mixed microbial consortia to produce PHA, an artificial microbial consortium can solve the limitation of the metabolic capacity of a single engineered strain and the low conversion efficiency of natural mixed microbial consortia.

**FIGURE 1:**
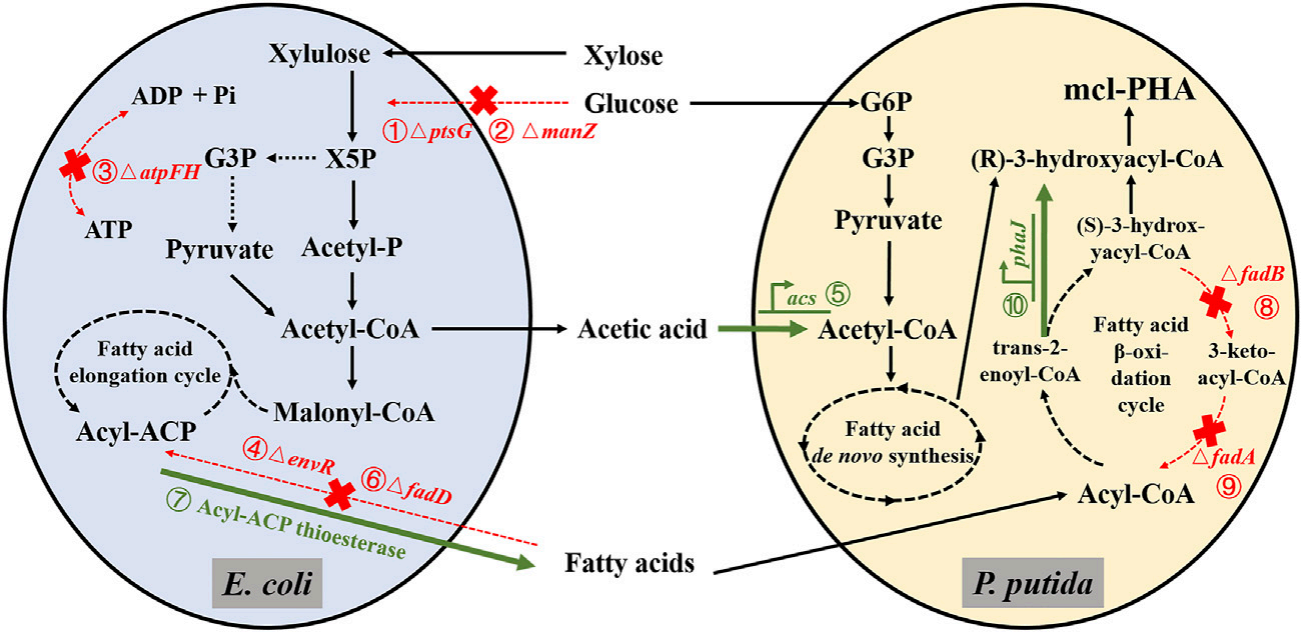
Schematic diagram of metabolic engineering for the production of mcl-PHA from a mixture of xylose and glucose by the microbial consortium based on the concept of “nutrient supply-detoxification” (Acronyms: X5P, Xylulose 5-phosphate; G3P, Glyceraldehyde-3-phosphate; Acetyl-P, Acetyl Phosphate; G6P, Glucose-6-Phosphate. The red crosses in the biosynthetic pathway indicate the deletion of corresponding genes. The bold green arrows indicate the overexpression of corresponding genes. The related genetic engineering represented by ①, ②, ③, and ④ was completed by (Liu et al., 2020), and the corresponding genetic engineering represented by ⑤ was completed by (Yang et al., 2019). The related genetic engineering represented by ⑥, ⑦, ⑧, ⑨, and ⑩ was conducted in this work).

In previous research, our laboratory used a modular construction strategy to develop a co-culture consortium composed of engineered *P. putida* KT2440 and *E. coli* MG1655 that can synthesize mcl-PHA from “unrelated” carbon sources (Yang et al., 2019; Liu et al., 2020). To avoid growth competition, the four genes *ptsG*, *manZ*, *atpFH,* and *envR* were knocked out in *E. coli* (shown as ①, ②, ③, and ④ in Figure 1) (Liu et al., 2020), the engineered *P. putida* KT2440 overexpressed the *acs* gene to promote the utilization of acetic acid and increase the accumulation of mcl-PHA (shown as ⑤ in Figure 1) (Yang et al., 2019). The microbial consortium composed of *E. coli* ∆4 and KT2440-acs efficiently utilized mixed sugars (glucose and xylose) and corn straw hydrolysate to produce mcl-PHA titers of 0.541 and 0.434 g/L, respectively (Liu et al., 2020). However, the conversion efficiency from the substrate to intermediate metabolites and final product was still limited, and the microbial consortium required further optimization.

In this work, we further optimized this previously developed microbial consortium to improve the production of mcl-PHA from xylose and glucose, “unrelated” carbon sources, by knocking out the *fadD* gene of *E. coli* while heterologously expressing the gene encoding the acyl carrier protein thioesterase from the castor plant (*Ricinus communis*), and knocking out the *fadA* and *fadB* genes in the β-oxidation pathway of *P. putida* while overexpressing the *phaJ* gene (shown as ⑥, ⑦, ⑧, ⑨, and ⑩ in Figure 1). Engineering *E. coli*, as the xylose-utilizing strain in our design, was capable of improved xylose utilization to produce acetic acid and FFAs, which act as intermediate metabolites of the microbial consortium. In the *P. putida*, we blocked a part of the β-oxidation pathway and improved the conversion efficiency of FFAs into mcl-PHA while relieving the growth inhibitory effects of acetic acid and FFAs on *E. coli*. Based on the above design, we reconstructed the artificial microbial consortium and then optimized its aerobic cultivation process, resulting in a significant improvement of the production of mcl-PHA and the efficiency of substrate conversion with mixed sugars (xylose and glucose). The successful construction of the microbial consortium based on the “nutrition supply–detoxification” concept provides insights for converting lignocellulose into high value-added compounds.

## Materials and Methods

### Bacterial Strains, Plasmids, and Reagents

The bacterial strains and plasmids used in this study are listed in Supplementary Table S1. *E. coli* MG1655 was donated by Dr. Tao Chen of Tianjin University, and *E. coli* ∆4 was stored in our laboratory. *P. putida* KT2440 (ATCC 47054) was obtained from the American type culture collection (Manassas, VA, United States). *E. coli* S17-1 was stored in our laboratory. The plasmid pBBR1MCS-2 was donated by Dr. Yingjin Yuan of Tianjin University, China. The plasmids pTKS/CS and pTKRED were donated by Dr. Tao Chen of Tianjin University. The plasmids pET28a and pK18mobsacB were stored in our laboratory. Xylose (99% purity) was purchased from Yuanye (Shanghai, China). Acetate (HPLC grade) was purchased from Concord Tech (China). Glucose (AR) was purchased from Yuanli Chemical (Tianjin, China). DNA manipulating agents, including restriction endonucleases and T4 DNA ligase, were purchased from Thermo Scientific (Beijing, China). Phanta Max Super-Fidelity DNA polymerase and Taq for polymerase chain reaction (PCR) were purchased from Vazyme (Nanjing, China). PCR primers were synthesized by GENEWIZ (Suzhou, China) and are listed in Supplementary Table S2.

### Plasmid and Strain Construction

*E. coli* MG1655 and *E. coli* ∆4 derivatives were used to construct the engineered strain for the biosynthesis of acetic acid and FFAs. According to the gene knockout method described previously (Lin et al., 2014), knockout of the *fadD* gene on the chromosomes of *E. coli* MG1655 and *E. coli* ∆4 through scarless chromosomal gene deletion strategy (Supplementary Figure S1). The primers tet-f and tet-r were used to amplify the tetracycline resistance gene and the I-SceI gene recognition sites at both ends, using pTKS/CS as the template. Using the genome of *E. coli* MG1655 as the template, the primer pairs *fadD* up-f/*fadD* up-r and *fadD* down-f/*fadD* down-r were used to amplify the 200 bp up and downstream flanking sequences of the *fadD* gene. The up-tet-down fragment was fused using overlap extension PCR and introduced into *E. coli* MG1655 and *E. coli* ∆4 by electrotransformation. The knockout strains *E. coli* ∆D and *E. coli* ∆4D were obtained after two rounds of resistance screening. Furthermore, pET28a was used as a vector to construct a heterologous expression vector using the T3 promoter and tac promoter to induce the expression of acyl carrier protein thioesterase. The original ricinoleoyl carrier protein sequence (GenBank: NM_001323748.1, Supplementary Appendix SA) was codon-optimized for *E. coli* (Supplementary Appendix SB) and synthesized by Genecreate (China). The primers RBS-ACP-f and ACP-r were used to amplify the ricinoleoyl carrier protein thioesterase gene fragment and add the ribosome binding site (RBS) in front of the fragment. The primers T3-ACP-f1/T3-ACP-f2/T3-ACP-f3 and ACP-r were then used to insert the T3 promoter and restriction sites on both sides of the sequence by multiplex PCR. Similarly, the primers tac-ACP-f1/tac-ACP-f2/tac-ACP-f3/tac-ACP-f4 and ACP-r were used for multiplex PCR to insert the tac promoter and restriction sites on both sides of the sequence. The amplified fragments were cloned into pET28a by restriction enzyme digestion and ligation to obtain the acyl carrier protein thioesterase expression vectors pET-T3-ACP and pET-tac-ACP, which were verified by DNA sequencing. The expression vectors were introduced into the target strain by electrotransformation to obtain the corresponding engineered strains.

*P. putida* KT2440 was used to construct the engineered strain for mcl-PHA synthesis, acetic acid, and FFAs utilization. Homologous recombination was used to knock out the *fadA* and *fadB* genes in the fatty acid β-oxidation pathway. The pK18mobsacB suicide plasmid was selected as the gene knockout vector, and the target gene was successfully knocked out through two rounds of homologous recombination. Using the genome of *P. putida* KT2440 as a template, specific primers were used to amplify 500 bp homology arms at the left and right ends of the gene to be knocked out. The homology arm fragments were ligated into the vector pK18mobsacB by restriction enzyme digestion and ligation to construct the knockout plasmids pK18-*fadA* and pK18-*fadB*, which were then transferred from *E. coli* S17-1 into *P. putida* KT2440 via intergeneric conjugation, and the knockout strain *P. putida* KT∆A was obtained through two homologous rounds of recombination and resistance screening. The same method was used to additionally knock out the *fadB* gene in *P. putida* KT∆A, resulting in the engineered strain *P. putida* KT∆AB. Furthermore, pBBR1MCS-2 was used to construct a vector for the co-expression of *acs* and *phaJ* genes using the T3 promoter and a synthetic RBS. Using *P. puti*da KT2440 as a template, the primer pairs acs-f/r and phaJ-f/r were used to amplify the *acs* and *phaJ* coding sequences, which were then cloned into pBBR1MCS-2 by restriction enzyme digestion and ligation to obtain p2*-acs-phaJ*. The expression vector p2-*acs*-*phaJ* was verified by restriction digestion and sequencing, and introduced into *P. putida* KT2440 and *P. putida* KT∆AB by electrotransformation, respectively.

### Culture Medium and Growth Conditions

Luria Bertani (LB) medium was used for strain preservation and seed culture preparation. All fermentation (aerobic fermentation) processes were carried out in M9 medium (12.8 g/L Na_2_HPO_4_ 7H_2_O, 3 g/L KH_2_PO_4_, 1 g/L NH_4_Cl, 0.5 g/L NaCl, and 0.24 g/L MgSO_4_), which was autoclaved and subsequently supplement with the required content of filter-sterilized glucose and/or xylose, and a trace element solution containing 6.0 mg/L FeSO_4_·7H_2_O, 2.7 mg/L CaCO_3_, 2.0 mg/L ZnSO_4_·H_2_O, 1.16 mg/L MnSO_4_·H_2_O, 0.37 mg/L CoSO_4_·7H_2_O, 0.33 mg/L CuSO_4_·5H_2_O, and 0.08 mg/L H_3_BO_3_. Where appropriate, 50 μg/ml kanamycin, 100 μg/ml chloramphenicol, and 2 mM Isopropyl β-d-1-thiogalactopyranoside (IPTG) were added. For the shake flask fermentations, single colonies of *E. coli* and *P. putida* were grown in 5 ml of culture medium in test tubes overnight at 30 °C and 220 rpm. The overnight cultures were used to inoculate 500 ml shake flasks containing 50 ml of M9 medium at a seed ratio of 1%, and fermented at 30°C and 220 rpm. For bioreactor fermentation, the overnight cultures were transferred at an inoculation ratio of 1%–100 ml of fresh LB medium and cultivated at 30°C and 220 rpm for 12 h to prepare the seed cultures. The seed cultures were used to inoculate a 5 L bioreactor (Bailun, Shanghai, China) with 2 L of M9 medium at a seed ratio of 0.5%, and the fermentation was carried out at 30 °C. Sterile air was delivered at a flow rate of two vvm, and the dissolved oxygen level was kept above 25% of air saturation by controlling the stirring speed. The pH was kept constant via the automatic addition of 0.5 M H_2_SO_4_ and 1.0 M KOH or 25% aqueous ammonia. Fed-batch strategies in bioreactor were performed to analyze the ability of engineered *P. putida* to produce mcl-PHA using acetic acid, with *P. putida* KT2440 as the control. The total concentration of acetic acid was quantified as 25 g/L through two feeds. Samples are taken at every interval to detect the concentration of acetic acid during the cultivation process to determine the feeding time. The fermentation experiments in the manuscript were conducted in triplicates, and data were shown as the mean values ± standard deviations (SD).

### Extraction and Analysis of Mcl-PHA

The extraction and analysis of PHA were described previously (Poblete-Castro et al., 2013). Briefly, an appropriate amount of culture solution was taken and the cells were harvested by centrifugation at 4°C and 8,000 rpm for 10 min and washed with distilled water. The washed bacteria were then frozen at −80°C and lyophilized for 24 h. Then, an appropriate amount of bacteria was weighed after drying, placed in a reactor containing 2 ml of esterification solution and 2 ml of chloroform, and esterified at 100°C for 4 h. The esterification solution was 3% sulfuric acid in methanol, and benzoic acid was added as an internal standard. After the esterification reaction was completed, pure water was added to the reaction mixture at room temperature, mixed evenly, and left standing for stratification. The lower organic phase was passed through a filter membrane to obtain the sample to be tested. The PHA was quantified by gas chromatography, with an injection volume of 1 µL. The starting temperature of the chromatographic separation column (Agilent HP-5) was 80°C and maintained for 1.5min, followed by ramp to 140°C at 30°C/min. Then the temperature was increased to 240°C at 40°C/min and kept at 240°C for 4 min, and the entire program lasted 10 min. The PHA monomer was characterized by gas chromatography-mass spectrometry. The starting temperature of the chromatographic separation column (Agilent HP-FFAP) was 50 °C and was maintained for 5 min. The temperature was then increased to 220°C at 5°C/min and kept for 20 min. The inlet temperature was 220°C, the ion source temperature was 230°C, and the interface temperature was 220°C.

### Analytical Methods

Cell optical density was measured at a wavelength of 600 nm (OD_600_) with UV-1200 spectrophotometer (Mapada, China). The OD_600_ value of two bacteria in the microbial consortium culture process was analyzed using the colony counting method (Liu X. et al., 2018; Liu et al., 2020). Based on the characteristics of kanamycin and chloramphenicol resistance in *E. coli* ∆4D (T3) and *P. putida* KTΔAB (p2-acs-phaJ), both *E. coli* ∆4D (T3) and *P. putida* KTΔAB (p2-acs-phaJ) are resistant to kanamycin, while *P. putid*a KTΔAB (p2-acs-phaJ) is also resistant to chloramphenicol. The first step is to test the quantitative relationship between the number of colonies of the two bacteria and their OD_600_, respectively. Diluting the bacterial liquids with different OD_600_ to the appropriate multiples and spreading them on the corresponding resistant plates, the quantitative relationships between OD_600_ and the number of colonies of the two bacteria was obtained as: for *E. coli* ∆4D (T3), 1OD_600_ = 0.42 × 10^9^ CFU/ml; for *P. putida* KTΔAB (p2-acs-phaJ), 1OD_600_ = 0.20 × 10^7^ CFU/ml. Secondly, the co-culture solutions of different periods were diluted to a certain multiple and then coated on plates with kanamycin and chloramphenicol resistance, respectively. The OD_600_ of the two bacteria in the co-culture can be calculated then, based on the different characteristics of resistance in *E. coli* ∆4D (T3) and *P. putida* KTΔAB (p2-acs-phaJ), and the above quantitative relationships.

Acetate, glucose, and xylose were quantified in the culture supernatant using an Ultimate 3000 HPLC (Dionex, Sunnyvale, CA, United States) equipped with an Aminex HPX-87H ion-exchange column (Bio-Rad, United States) operating at 65 °C and a differential refraction detector, with 5 mM H_2_SO_4_ as the mobile phase at a flow rate of 0.6 ml/min. Extraction and detection of fatty acids were performed as described previously (Eiteman and Altman, 2006). Briefly, 2 ml of fermentation broth was centrifuged at 12,000 rpm for 5 min, and the supernatant was collected. Then, 200 μL of glacial acetic acid and 150 mg of internal standard (undecanoic acid) were added to the supernatant, mixed with 2 ml of extractant (n-hexane: chloroform = 4: 1 v/v), and shaken thoroughly. After standing still, the above-mentioned mixed solution was placed in the inner lining of the reactor, and allowed to stand overnight in a fume hood. After the organic reagents were completely volatilized, 1 ml of esterification solution (chloroform: methanol: sulfuric acid = 10: 8.5: 1.5, v/v) was added, the reactor was sealed and placed in an oven at 100 °C for 1 h. After the reaction was completed, pure water was added to the reaction mixture at room temperature, mixed evenly, and left standing for stratification. The lower organic phase was passed through a filter membrane to obtain the sample to be tested. The fatty acids were quantified by gas chromatography, with an injection volume of 1 µL. The starting temperature of the chromatographic separation column (Agilent HP-5) was 60 °C and maintained for 3min. Then the temperature was increased to 250 °C at 10°C/min and kept at 250 °C for 10 min, and the entire program lasted 30 min.

## Results and Discussion

### The Division of Labor and Collaborative Design of the Artificial Microbial Consortium

Previous studies have shown that adding fatty acid as external substrates will significantly increase the titer of mcl-PHA synthesis by *P. putida* and the final yield (Wang and Nomura, 2010). As FFAs are the preferred carbon source for *P. putida* to synthesize mcl-PHA, their toxicity to cells and production costs must be considered as a priority. Therefore, we aimed to start the synthesis from the main sugars in the cheap substrate cellulosic hydrolysate, such as xylose and glucose. However, pure cultures of *P. putida* cannot co-utilize glucose and xylose to produce mcl-PHA. To overcome this, we introduced an engineered *E. coli* in which the four genes *ptsG*, *manZ*, *atpFH,* and *envR* were knocked out, while further strengthened its functions, and co-cultured it with engineered *P. putida* to form a microbial consortium to produce mcl-PHA from glucose and xylose. As shown in Figure 1, based on the construction principle of division of labor, when synthetic co-cultures are used to perform complex tasks, the functions of the two strains in the microbial consortium are distinguished. The two strains are independent but play complementary roles in the microbial consortium, which we call “nutrition supply–detoxification” (Liu et al., 2020). We further strengthened this interaction by designing an engineered *E. coli* with increased secretion of long-chain FFAs. Then, by weakening the β-oxidation of fatty acids in *P. putida* while strengthening its acetic acid utilization capacity, it was able to efficiently produce mcl-PHA. As for the co-culture design, we conducted the performance tests on all engineered *P. putida* and engineered *E. coli* after each step of genetic modification, which indirectly reflects the effect of each genetic operation on the synthesis of the target product. The optimized microbial consortium based on the “nutrition supply–detoxification” concept is expected to enable the industrial production of mcl-PHA from glucose-xylose mixtures.

### The Performance of Engineered *E. coli* in the Production of Acetic Acid and FFAs

#### Engineering *E. coli* for the Production of Acetic Acid and FFAs

Unlike most strains that naturally produce fatty acids, *E. coli* usually does not accumulate fatty acids in the cell. In the fatty acid metabolic pathway of *E. coli*, the *fadD* gene encodes a long-chain fatty acid coenzyme A ligase that plays a vital role in the activation and secretion of fatty acids. Voelker and Davies found that engineering *E. coli* MG1655 with a mutation in the *fadD* gene was significantly different from other strains in terms of FFA titer and type, producing up to 2.0 g/L of FFAs in shake-flask culture (Voelker and Davies, 1994). Therefore, we knocked out the *fadD* gene in wild-type *E. coli* MG1655 and engineering *E. coli* Δ4, resulting in the strains *E. coli* ΔD and engineering *E. coli* Δ4D respectively. Li et al. further modified the MG1655 mutant strain by knocking out the *fadD* gene and simultaneously expressing an exogenous acyl sulfide lipase gene (Li et al., 2012). The resulting strain was able to use xylose as the sole carbon source to produce 2.62 g/L of FFAs. The lack of an acyl carrier protein thioesterase gene in *E. coli* results in poor secretion of fatty acids. The transfer of acyl carrier protein thioesterase into *E. coli* can disrupt the elongation cycle of fatty acids and promote the extracellular secretion of fatty acids. Studies have confirmed that acyl carrier protein thioesterases can be divided into FatA and FatB thioesterase families according to their substrate specificity. FatA thioesterase has activity on unsaturated acyl carrier protein, while FatB thioesterase has stronger activity on saturated acyl carrier protein (Salas and Ohlrogge, 2002). In addition, acyl carrier protein thioesterases from different sources have different activities on acyl carrier proteins with fatty acids of varying chain lengths (Yuan et al., 1995). Zhang et al. confirmed that heterologous expression of the acyl carrier protein thioesterase gene from the castor plant (*Ricinus communis*) in *E. coli* can increase the production of extracellular FFAs to 2.0 g/L (Zhang et al., 2011). We codon-optimized the acyl carrier protein thioesterase gene sequence from the castor plant and used a T3 promoter and tac promoter to induce expression, respectively.

**FIGURE 2:**
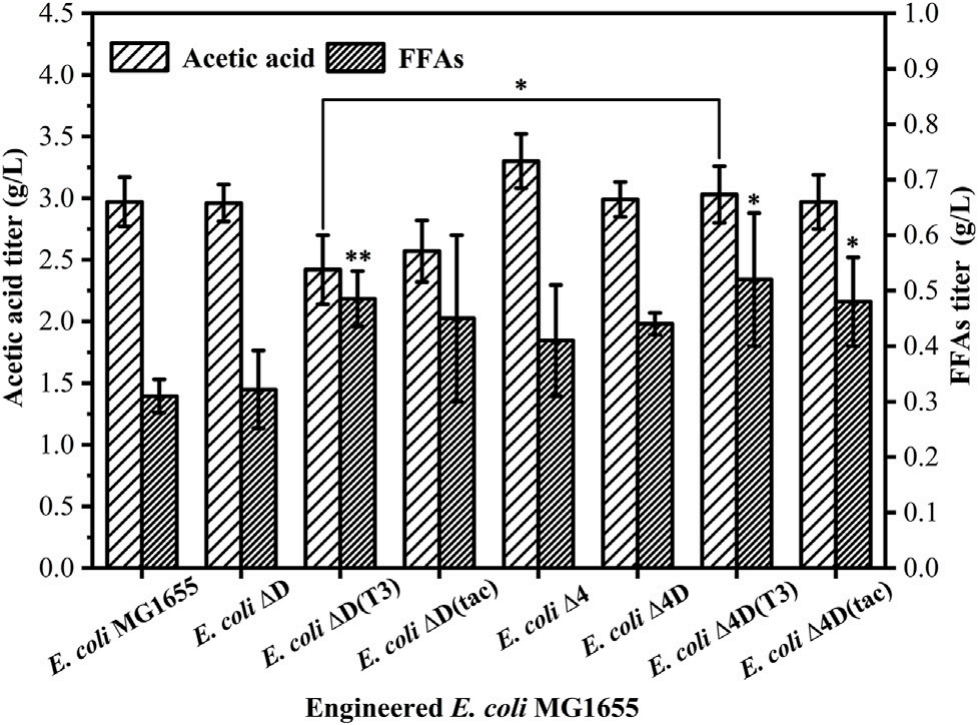
Comparison of the ability of engineered *E. coli* to produce acetic acid and FFAs in shake flasks, and the cultivation time was 60 h. The error bars indicate the standard deviation of triplicate experiments. *p < 0.05; **p < 0.01; ***p < 0.001; ns, no significance.

Preliminary tests on acetic acid and FFAs production in engineered *E. coli* and the parental strain (Figure 2) showed the strains engineered based on *E. coli* Δ4 have a stronger ability to produce acetic acid than *E. coli* MG1655. As expected, the strain with only the *fadD* gene knocked out showed the same result. In a comprehensive comparison, the engineered strains with *fadD* gene knockout and expression of the exogenous acyl carrier protein thioesterase gene produced more FFAs. *E. coli* Δ4D (T3) and *E. coli* Δ4D (tac) produced about 3.0 g/L acetic acid and 0.5 g/L FFAs. There is no statistical difference as *E. coli* MG1655 as the control, so the degree of enhancement was not ideal. By comparing the OD_600_ of *E. coli* MG1655 and engineered strains at the end of cultivation, it was found that the OD_600_ of *E. coli* MG1655 was 2.29, while the OD_600_ of *E. coli* Δ4D (T3) and *E. coli* Δ4D (tac) was only 1.62 and 1.69 respectively (Supplementary Figure S2). Hence, the growth of the engineered strains was worse than that of wild-type bacteria. At the same time, we measured the residual amount of xylose in the fermentation broth, and found that the five strains *E. coli* MG1655, *E. coli* ΔD, *E. coli* ΔD (T3), *E. coli* ΔD (tac), and *E. coli* Δ4 completely consumed xylose, while *E. coli* Δ4D, *E. coli* Δ4D (T3), and *E. coli* Δ4D (tac) still had approximately 2.8 g/L of residual xylose (Supplementary Figure S2). The excessive accumulation of acetic acid may have an inhibitory effect on the fermentation system of the strain. On the one hand, acetic acid will cause the pH to drop and thus form an inhibitory effect. On the other hand, when the pH is close to the pKa of acetate (approximately 4.76), more of the acetate would be in its protonated form so that toxic concentrations of acetic acid itself may cause more potent inhibition. We hypothesized that the accumulation of acetic acid produced by the strains in the shake flask cultivation system caused the system’s pH to decrease, inhibiting the growth of the bacteria and the utilization of xylose. The pH of the cultivation broth was tested, and the pH was around 4.65, which partially confirmed our hypothesis and maybe explained why the engineered strain was unable to reach its full potential in the shake flask cultivation system.

#### Fermentation of Engineered *E. coli* to Produce Acetic Acid and FFA

**FIGURE 3:**
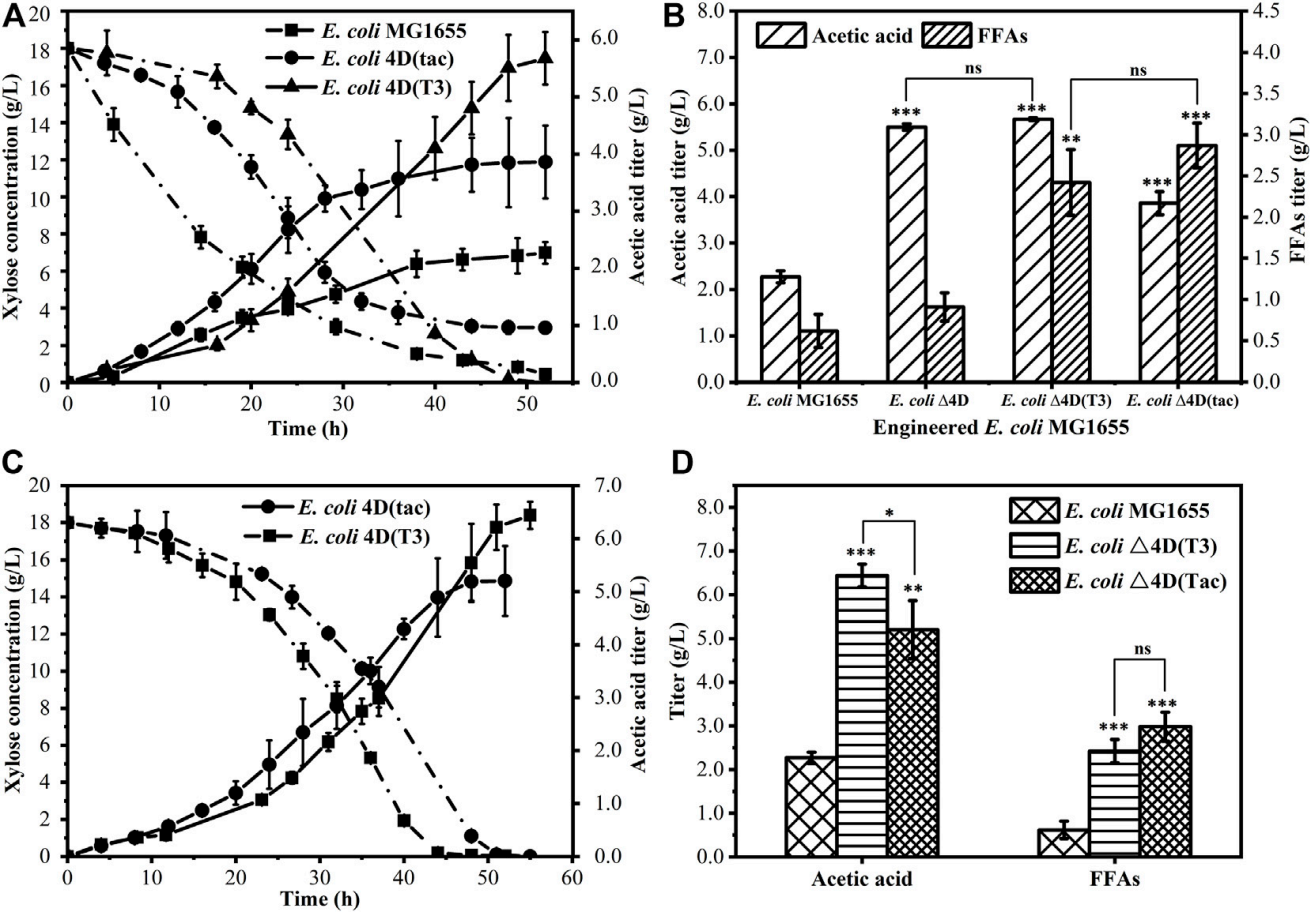
Production of acetic acid and fatty acids by engineered *E. coli* in a bioreactor **(A)** Process diagram of *E. coli* MG1655, *E. coli* Δ4D (T3), and *E. coli* Δ4D (tac) using xylose to produce acetic acid in a bioreactor **(B)** acetic acid and FFAs titer obtained by fermentation of *E. coli* MG1655 and engineered *E. coli*, and the cultivation time was 55 h **(C)** Process diagram of *E. coli* Δ4D (T3) and *E. coli* Δ4D (tac) using xylose to produce acetic acid in a bioreactor after optimization of the nitrogen source **(D)** Comparison of the titers of acetic acid and FFAs obtained by fermentation of *E. coli* MG1655 and engineered *E. coli*, and the cultivation time was 55 h. The error bars indicate the standard deviation of triplicate experiments. *p < 0.05; **p < 0.01; ***p < 0.001; ns, no significance.

Bioreactors can be used to control the dynamic balance of pH in the fermentation system, effectively avoiding the negative effects of excessive acetic acid accumulation in the fermentation environment, which leads to a decrease in pH and inhibits bacterial growth. As shown in Figure 3A, during the same fermentation time, the wild-type bacteria initially consumed xylose quickly, which may be caused by the excessive growth of the initial biomass of the wild-type bacteria. By contrast, *E. coli* Δ4D (T3) and *E. coli* Δ4D (tac) showed faster xylose consumption in the middle of the fermentation, and at this time, the production of acetic acid was also quicker. The output of FFAs and acetic acid at the end of the fermentation is shown in Figure 3B. The acetic acid output of *E. coli* MG1655 was 2.27 g/L, while *E. coli* Δ4D (T3) and *E. coli* Δ4D (tac) produced 5.67 and 3.86 g/L of acetic acid, respectively. It can be seen that the engineered strain *E. coli* Δ4D (T3) has an outstanding ability to produce acetic acid. However, *E. coli* Δ4D (tac) and *E. coli* Δ4D (T3) produced 2.87 g/L and 2.42 g/L FFAs, respectively. Compared with *E. coli* MG1655, the corresponding titers were increased 4.6 and 4.0 times. Consistent with previous studies, heterologous expression of the acyl carrier protein thioesterase gene of the castor plant in *E. coli* enhanced its ability to secrete FFAs outside the cell.

The engineered strains *E. coli* Δ4D (T3) and *E. coli* Δ4D (tac) were compared with *E. coli* MG1655 in the bioreactor fermentation to obtain acetic acid and FFAs, the production was greatly improved. This confirmed the effect of bacterial growth on the production of acetic acid and FFAs. From the perspective of the culture medium, the limitation of nitrogen source is an essential factor affecting the growth of bacteria. We changed the pH-adjusting solution from 1M KOH to 25% aqueous ammonia to provide sufficient nitrogen. As shown in Figure 3C, the engineered strain’s xylose consumption phase was shortened after switching of pH control, while the titer of acetic acid obtained by fermentation was increased. At the end of fermentation, the acetic acid output of *E. coli* Δ4D (T3) and *E. coli* Δ4D (tac) was raised to 6.44 and 5.20 g/L, respectively, while the FFAs yields were similar to those obtained before optimization (Figure 3D). The main extracellular FFAs included n-hexanoic acid, n-heptanoic acid, n-octanoic acid, n-decanoic acid, and n-dodecanoic acid. The switching of pH control improved the growth of engineered strains in the fermentation process, which accelerated the degradation of xylose and its conversion into acetic acid. These results show that both engineered *E. coli* Δ4D (T3) and *E. coli* Δ4D (tac) can efficiently use xylose to produce acetic acid and FFAs. After switching of pH control, the acetic acid production of *E. coli* Δ4D (T3) was 3.0 times higher than that of wild-type bacteria, and the output of FFAs was about 4.0 times higher than that of wild-type bacteria. However, *E. coli* Δ4D (tac) requires the addition of IPTG as inducer during the fermentation process, and considering the limitations of adding IPTG during large-scale fermentation and its effect on the fermentation costs, the engineered strain *E. coli* Δ4D (T3) was a better option for constructing the microbial consortium.

### Engineering *P. putida* to Produce Mcl-PHA

#### Engineered *P. putida* Uses Acetic Acid to Accumulate Mcl-PHA

Based on the collaborative design of the microbial consortium for mutual benefit and symbiosis, the available carbon sources of *P. putida* include the initial substrate glucose, as well as the acetic acid and FFAs provided by the engineered *E. coli*. As “unrelated” carbon sources, glucose and acetic acid can be converted into mcl-PHA through the fatty acid synthesis pathway via acetyl-CoA as an immediate precursor. Therefore, strengthening the acetic acid assimilation pathway and promoting its conversion into acetyl-CoA can encourage the accumulation of mcl-PHA. In the previous study by (Yang et al., 2019), overexpression of the *acs* gene in *P. putida* KT2440 increased the strain’s ability to utilize acetate and promoted the conversion of acetate into mcl-PHA. Therefore, we also overexpressed the *acs* gene. FFAs are “related” carbon sources for the synthesis of mcl-PHA through fatty acid β-oxidation in *P. putida*. Weakening the fatty acid β-oxidation pathway can redirect the carbon flux toward PHA precursors, which is beneficial to increase the production of mcl-PHA (Salvachua et al., 2020; Zhao et al., 2020). Studies have confirmed that knocking out the *fadA* and *fadB* genes in the fatty acid β-oxidation pathway of *P. putida* leads to the conversion of most fatty acids into 3-hydroxyacyl-CoA for the synthesis of PHA, rather than them being oxidized into acetyl-CoA, thereby significantly improving the conversion rate of the substrates into mcl-PHA (Wang et al., 2009; Chung et al., 2011; Liu et al., 2011). Therefore, the two previously identified genes encoding 3-hydroxyacyl-CoA dehydrogenase (*fadB*) and 3-ketoacyl-CoA thiolase (*fadA*) were knocked out in *P. putid*a KT2440, resulting in engineered strain KTΔAB. On the other hand, the relationship between fatty acid β-oxidation and PHA synthesis can still be established through enoyl-coenzyme A hydratase (PhaJ). In fatty acid β-oxidation, PhaJ can hydrate the intermediate 2-trans-enoyl-CoA to generate the mcl -PHA precursor (R)-3-hydroxyacyl-CoA. The overexpression of the *phaJ* gene in *P. putida* leads to an increase in the accumulation of mcl-PHA, illustrating the critical role of this enzyme in linking fatty acid β-oxidation with PHA synthesis from “related” carbon sources.

**FIGURE 4:**
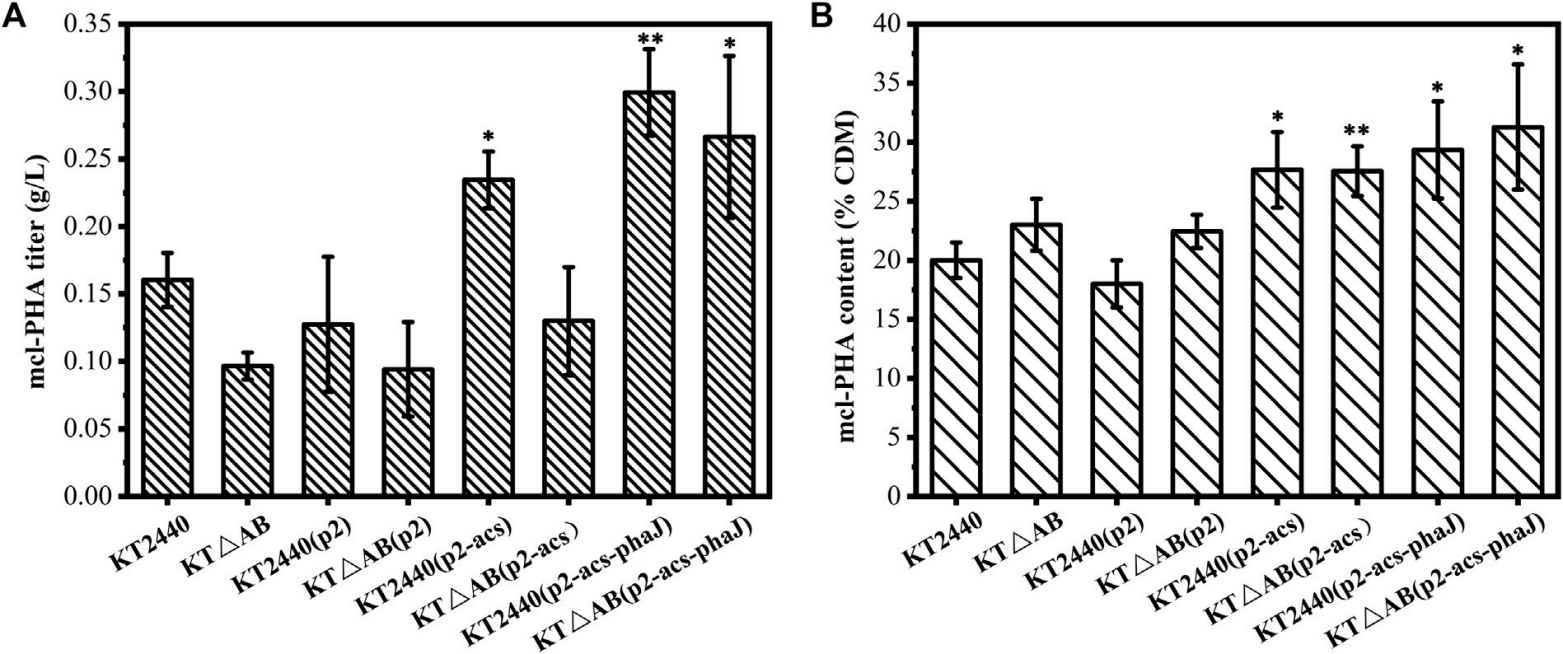
Comparison of the production of mcl-PHA by engineered *P. putida* in shake-flask fermentation with acetic acid as the sole carbon source, and the cultivation time was 60 h **(A)** The titer of mcl-PHA **(B)** Intracellular content of mcl-PHA. The error bars indicate the standard deviation of triplicate experiments. *p < 0.05; **p < 0.01; ***p < 0.001; ns, no significance.

The mcl-PHA production capacity of the engineered *P. putida* was tested with acetic acid as the sole source in shake flasks. As shown in Figure 4A, the engineered strains *P. putida* KT2440 (p2-acs-phaJ) and *P. putida* KTΔAB (p2-acs-phaJ) showed efficient utilization of acetic acid and accumulated 0.30 and 0.27 g/L of mcl-PHA. Compared with the production of mcl-PHA by *P. putida* KT2440 (0.16 g/L), this is a dramatic improvement. Thus, the overexpression of the *acs* and *phaJ* genes increased the output of mcl-PHA by the engineered strains, which was consistent with previous studies. The intracellular content of mcl-PHA in the engineered strains in which the *fadA* and *fadB* genes were knocked out was higher. *P. putida* KTΔAB (p2-acs-phaJ) accumulated mcl-PHA equivalent to 31.27% of the dry cell mass (CDM), which was significantly higher than in *P. putida* KT2440 (Figure 4B). However, the gene knockout caused a delay in the biomass accumulation of the strain. The consequent reduction of biomass resulted in a slightly lower final mcl-PHA titer compared to the engineered strains without a knock out of the *fadA* and *fadB* genes. Similarly, the metabolic burden stemming from the propagation of plasmid vectors will slightly reduce the accumulation of mcl-PHA indicating that the biomass of engineered strains is highly correlated with the accumulation of mcl-PHA.

**FIGURE 5:**
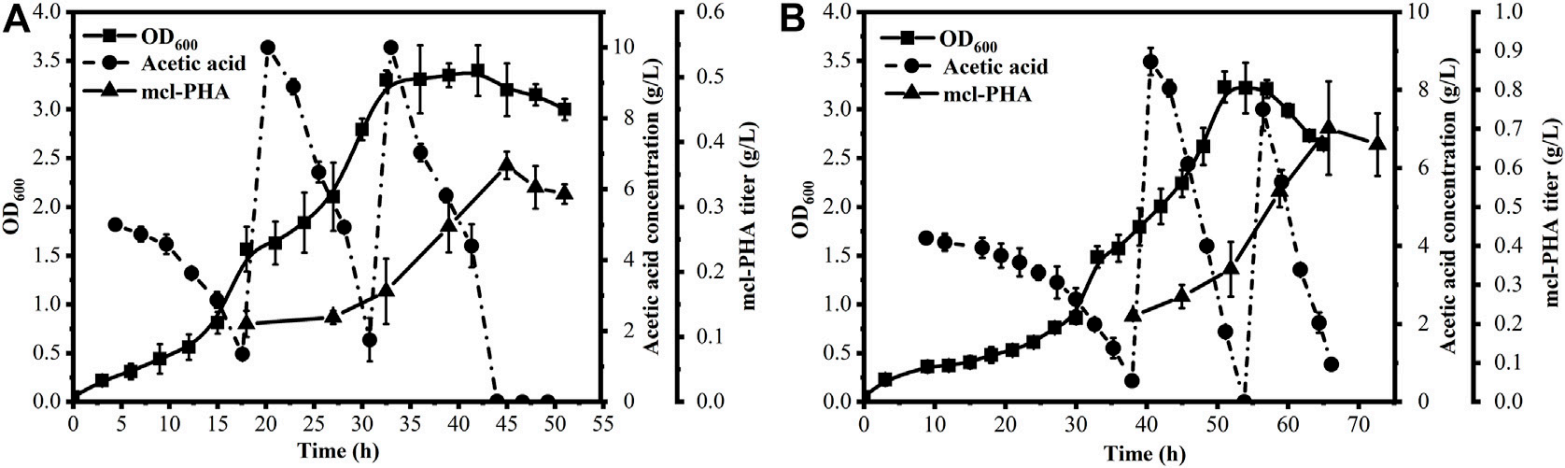
*P. putida* KT2440 **(A)** and the engineered strain *P. putida* KTΔAB (p2-acs-phaJ) **(B)** were used to produce mcl-PHA from acetic acid as the sole substrate in a bioreactor through fed-batch strategy. The error bars indicate the standard deviation of triplicate experiments.

A comparative experiment was conducted in a bioreactor to reduce the environmental impact such as oxygen limitation and the inhibitory effect of acetic acid on the biomass of the strains. *P. putida* KT2440 and *P. putida* KTΔAB (p2-acs-phaJ) were grown with acetic acid as the sole carbon source for fed-batch fermentation. By detecting the concentration of acetic acid in the system, *P. putida* KT2440 was fed with acetic acid at 15 and 30 h, and *P. putida* KTΔAB (p2-acs-phaJ) was fed at 33 and 51 h. As shown in Figure 5, *P. putida* KTΔAB (p2-acs-phaJ) exhibited a lag in biomass accumulation compared with *P. putida* KT2440, so the fermentation time was 5 h longer than in *P. putida* KT2440. However, the biomass obtained by the two strains by the end of the bioreactor fermentation was similar. The intracellular PHA content of *P. putida* KTΔAB (p2-acs-phaJ) (43.48% of CDM) was twice that of *P. putida* KT2440 (21.81% of CDM). The final titer of mcl-PHA was 0.70 g/L, which was twice that of *P. putida* KT2440. Therefore, a series of genetic modifications of *P. putida* greatly increased its ability to use acetic acid to produce mcl-PHA. In addition, *P. putida* KTΔAB (p2-acs-phaJ) has similar yields (about 0.03 g/g) of mcl-PHA obtained by using the different concentration of acetic acid in the shake flask (Figure 4A) and the bioreactor (Figure 5), which may indicate that the limitation of oxygen in shake flask culture has less influence on the accumulation of mcl-PHA.

#### Engineering *P. putida* to Produce Mcl-PHA by Fermentation With Mixed Substrates

**FIGURE 6:**
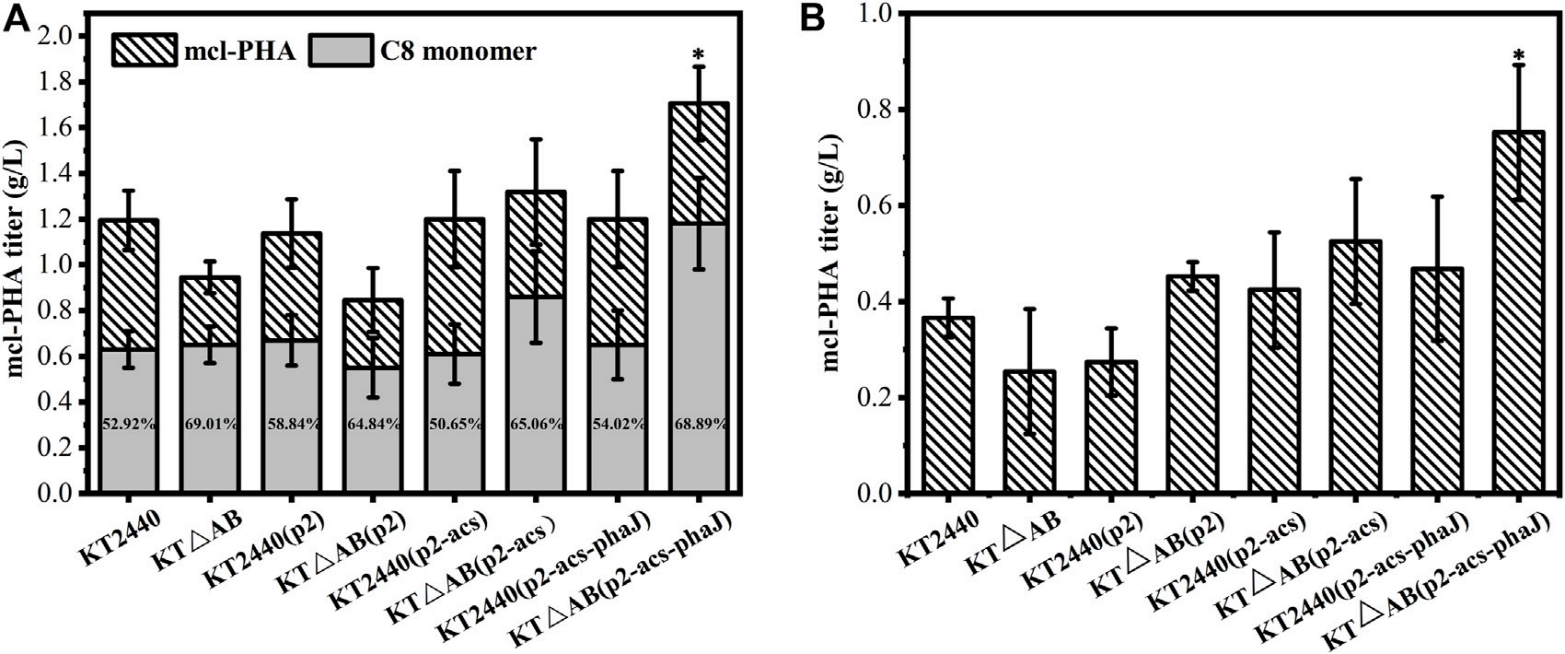
The engineered strain *P. putida* KTΔAB (p2-acs-phaJ) was used to produce mcl-PHA by fermentation with mixed substrates **(A)** *P. putida* KTΔAB (p2-acs-phaJ) was used to produce mcl-PHA in a two-step fermentation with a mixed substrate comprising 10 g/L glucose and 2 g/L octanoate **(B)** *P. putida* KTΔAB (p2-acs-phaJ) was cultured with 5 g/L glucose and 5 g/L acetic acid as a substrate for 24 h, after which 2 g/L octanoate was added, and the fermentation was continued for 72 h to measure the final mcl-PHA titer. The error bars indicate the standard deviation of triplicate experiments. *p < 0.05; **p < 0.01; ***p < 0.001; ns, no significance.

Previous studies have shown that FFAs are the preferred substrate of *P. putida* to achieve large amounts of PHA accumulation in the cell. The PHA polymer exhibits an even-numbered carbon chain length unless there are odd-chain length fatty acids in the substrate (Ma et al., 2009), which illustrates the critical role of fatty acid β-oxidation in the synthesis of mcl-PHA by *P. putida*. *P. putida* KT2440 could degrade octanoate into acyl-CoA and use the β-oxidation pathway to synthesize C6 or C8 R-3-hydroxyacyl-CoA as the β-oxidation intermediates, which are mcl-PHA precursors. While knocking out the *fadA* and *fadB* genes weakens the β-oxidation pathway, it promotes C8 R-3-hydroxyacyl-CoA to become more mcl-PHA precursors. Therefore, the content of the C8 monomer in mcl-PHA accumulated by engineered *P. putida* using octanoate may be higher than that of *P. putida* KT2440. In the microbial consortium, engineered *E. coli* provides *P. putida* with the optimal metabolites for accumulation of mcl-PHA that medium- and long-chain fatty acids. We observed the utilization of FFAs by engineered *P. putida* in a two-step fermentation with glucose and octanoate are the only carbon sources in the medium. As shown in Figure 6A, the mcl-PHA titer of the engineered *P. putida* with a knockout of the β-oxidation-related genes was lower than that of *P. putida* KT2440, which is suspected to be related to growth inhibition. Ultimately the mcl-PHA titer of the fully engineered strain *P. putida* KTΔAB (p2-acs-phaJ) reached 1.71 g/L. The synthesized mcl-PHA monomer is composed of 3-hydroxyhexanoate (C6), 3-hydroxyoctanoate (C8), 3-hydroxydecanoate (C10), 3-hydroxydodecanoate (C12), and 3-hydroxytetradecanoate (C14) (Supplementary Table S3). Predictably, octanoate causes the C8 monomer to become the main component of the polymer composition. Further comparing the C8 monomer content of mcl-PHA, the average C8 monomer in mcl-PHA of the engineered strains with modified β-oxidation related genes accounted for 66.95% of the total compared to the unmodified engineered strains (54.11%). Similarly, the C8 monomer in the mcl-PHA accumulated by *P. putida* KTΔAB (p2-acs-phaJ) accounted for 68.89% of the total. Consistent with our expectations, modification of the β-oxidation pathway can improve the conversion efficiency of FFAs to synthesize mcl-PHA precursors and then mcl-PHA. To test the engineered *P. putida* to produce mcl-PHA in the microbial consortium, the substrate utilization mechanism of the microbial consortium was simulated at the shake flask level. Acetic acid was added as one of the mixed substrates based on the above research. As shown in Figure 6B, the engineered strain *P. putida* KTΔAB (p2-acs-phaJ) was able to produce 0.75 g/L mcl-PHA, which was twice that of *P. putida* KT2440 (0.37 g/L). It can be seen that the engineered *P. putida* can effectively use the mixed substrates of the microbial consortium to produce mcl-PHA, which includes glucose, as well as the acetic acid and FFAs secreted by *E. coli*.

### Construction of the Microbial Consortium to Produce Mcl-PHA From a Glucose-Xylose Mixture

**FIGURE 7:**
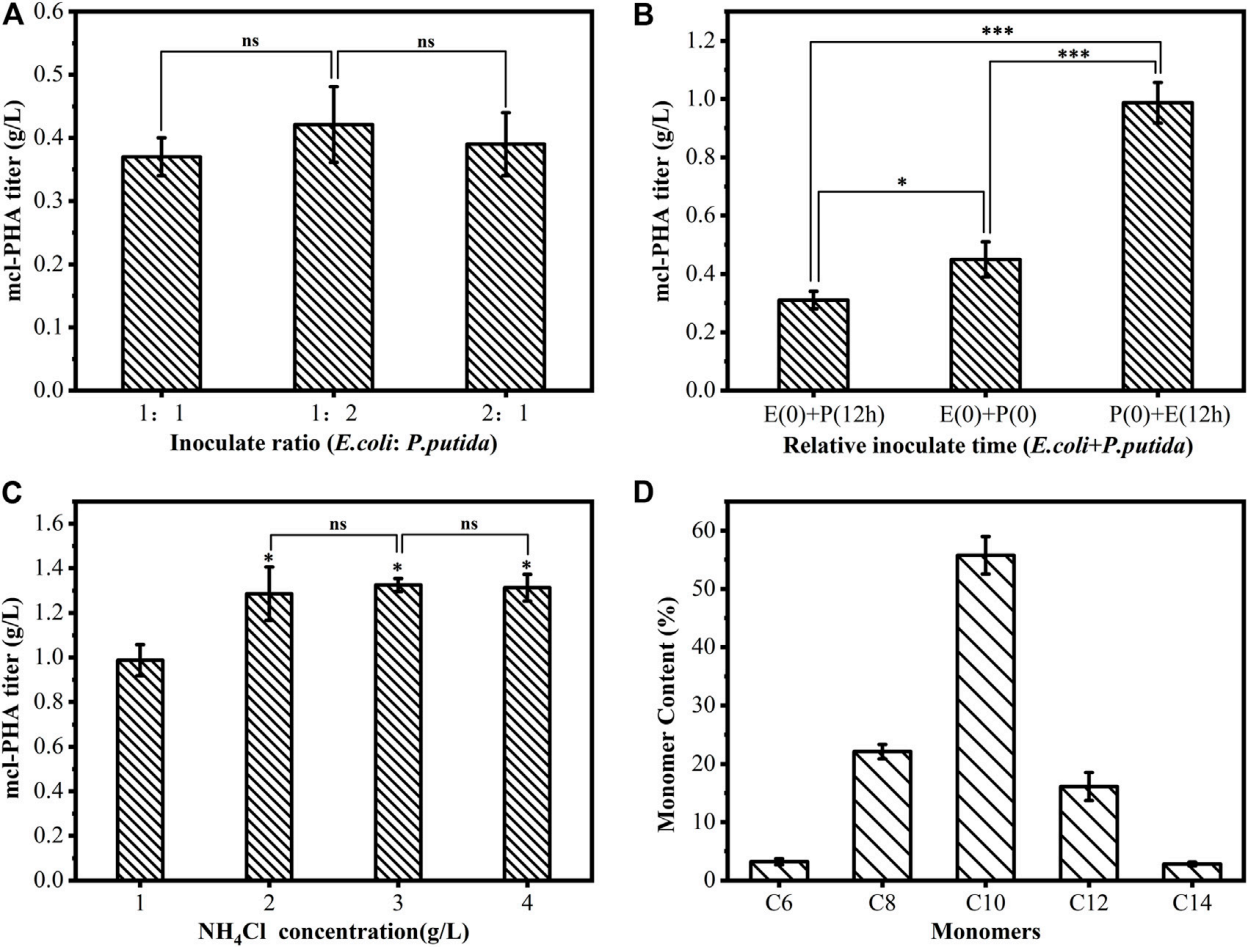
The artificial microbial consortium with optimized inoculation ratio, inoculation time, and nitrogen source was used to convert a mixture of glucose and xylose (mass ratio = 1:1) into mcl-PHA in the bioreactor, and the cultivation time was 60 h **(A)** Different inoculation ratios (vol/vol) **(B)** Different relative inoculation times; E (0) + P (12 h), *P. putida* inoculated 12 h after inoculation with *E. coli*; E (0) + P (0), Simultaneous inoculation of *E. coli* and *P. putida*; P (0) + E (12 h), *E. coli* inoculated 12 h after inoculation with *P. putida* **(C)** Different nitrogen source concentrations **(D)** The monomer composition of mcl-PHA produced by the microbial consortium. The error bars indicate the standard deviation of triplicate experiments. *p < 0.05; **p < 0.01; ***p < 0.001; ns, no significance.

The microbial consortium uses xylose and glucose, the main components of cellulosic hydrolysate, as substrates to produce mcl-PHA. Based on our design principle, two engineered strains were designed and extensively tested to construct a mutually beneficial symbiosis. Ideally, *E. coli* Δ4D (T3) in the microbial consortium should preferentially metabolize xylose, and *P. putida* KTΔAB (p2-acs-phaJ) metabolizes glucose to avoid substrate competition, thereby ensuring the stability of the microbial consortium. However, the artificial microbial consortium for the production of mcl-PHA is dynamic. Coordinating the interactions in the microbial consortium and the synergistic growth of the two strains is essential for optimizing the co-culture strategy. Therefore, the relative inoculation ratio (the volume ratio of the seed liquid), inoculation time, and nitrogen source concentration of the microbial consortium were optimized. Firstly, the two strains in the microbial consortium were added at different inoculation ratios (*E. coli*: *P. putida =* 1:1, *E. coli*: *P. putida =* 1:2, *E. coli*: *P. putida =* 2:1). As shown in Figure 7A, at an *E. coli*: *P. putida* ratio of 1:2, the maximum mcl-PHA titer produced by the microbial consortium was 0.42 g/L. However, the mcl-PHA titers were not statistically different under different inoculation ratios, which may be that the relatively fast-growing strains quickly reached equilibrium in the co-culture. According to the plate counting assay, *E. coli* was the dominant strain in the microbial consortium at the end of the fermentation at three different inoculation ratios. Because mcl-PHA is an intracellular product, the biomass accumulation of *P. putida* may affect the production of mcl-PHA in co-culture. If *P. putida* becomes the dominant strain in the co-culture system, it will increase the output of mcl-PHA. Therefore, the glucose and xylose consumption was measured in the microbial consortium with an *E. coli*: *P. putida* inoculation ratio of 1:2. As shown in Supplementary Figure S3, all glucose was consumed after 20 h of fermentation, with 6.24 g/L residual xylose remaining at this time. This result indicated that there may still be carbon catabolite repression and competition between the two strains for glucose in the co-culture. In the microbial consortium, *E. coli* Δ4D (T3) prefers to metabolize xylose but still consumes glucose, which causes the glucose to be consumed first, and *E. coli* becomes the dominant strain in the microbial consortium by the end of the fermentation.

The relative inoculation time of the two strains (*E. coli* was added to the fermentation medium 12 h before *P. putida* inoculation, 12 h after *P. putida* inoculation, and at the same time with *P. putida* inoculation) may have a significant impact on the microbial consortium. As shown in Figure 7B, the co-culture system that was first inoculated with *E. coli* produced 0.31 g/L of mcl-PHA, while the co-culture system in which *E. coli* was added to the medium 12 h after *P putida* inoculated produced 0.99 g/L of mcl-PHA. The results of plate colony counting showed that the adjustment of the relative inoculation time of the two strains reversed the status of the dominant strain in the microbial consortium. Thus, inoculation with *P. putida* before adding *E. coli* avoids the competition between the two strains for glucose. It promotes *P. putida* to become the dominant strain in the co-culture system while also weakening the inhibitory effect of acetic acid and FFAs secreted by *E. coli* into the co-culture system on its own growth. The two strains grew cooperatively, and the mcl-PHA output of the system was increased.

*P. putida* in the microbial consortium is a functional strain that produces mcl-PHA. *P. putida* accumulates mcl-PHA in the cell under the conditions of limited nitrogen and abundant carbon sources. However, supplementing with good nitrogen sources is beneficial to the growth of *E. coli* and the secretion of acetic acid and FFAs. Based on the difference in the nitrogen source requirements of the two strains in the microbial consortium, the concentration of the nitrogen source in the medium of the co-culture system was optimized. As shown in Figure 7C, when the concentration of NH_4_Cl reached 3.0 g/L, the co-culture system produced 1.32 g/L of mcl-PHA and a further increase of the NH_4_Cl concentration did not increase the output of mcl-PHA. However, when the nitrogen source concentration was 1.0 g/L, there may be nitrogen limitation, which caused the mcl-PHA titer to be lower than that at other nitrogen source concentrations. The monomer composition analysis of mcl-PHA produced by the microbial consortium is shown in Figure 7D. The polymer contained C6, C8, C10, C12, and C14 monomers, whereby the C14 monomers were partially unsaturated, and C10 monomers were the most abundant, accounting for 55.76% of the total content of mcl-PHA. In addition to mcl-PHA containing monomers with 6–14 carbon atoms, it also contains small amounts of C16 and C16:1 unsaturated long-chain monomers. These lcl-monomers were not observed in the pure culture of *P. putida*, which may be caused by the engineered *P. putida* using undetected lcl-FFAs secreted by *E. coli* to form lcl-PHA monomers. The microbial consortium based on the concept of “nutrient supply-detoxification” can therefore effectively use the “unrelated” carbon sources glucose and xylose to produce mcl-PHA by coordinating the metabolic communication and interaction between the two strains.

## Perspectives

From the perspective of synthetic biology goals and tasks, artificial microbial consortia are suitable for more complex tasks and are more robust to environmental changes than pure cultures. At present, the relevant research on the synthesis of PHA by microbial consortia is mostly focused on the screening of unique microorganisms that can cooperate with each other to accumulate PHA using cheap substrates. However, most of them produce scl-PHA or copolymers (Table 1). Rebocho et al. co-cultured *Cupriavidus necator* DSM 428 and *Pseudomonas citronellolis* NRRL B-2504, using apple pulp extract rich in sugars such as fructose and glucose as the substrate, which resulted in a PHA blend with a titer of 1.85 g/L, containing about 48 wt% of P (3HB) and 52 wt% of mcl-PHA (Rebocho et al., 2020). Generally, the synthesis of mcl-PHA is highly dependent on the substrate. Although a single metabolically engineered strain can increase the titer, it still must be supplied with FFAs rich substrates, such as waste oil or simple FFAs. In addition to its own substrate cost, it is limited by the strain’s ability to decompose fats and the robustness of production during large-scale fermentation. Microorganisms in nature often adapt to complex environments by forming interactive symbiotic communities and the division of labor between strains (Agapakis et al., 2012). Targeted design and construction of microbial consortia can achieve tasks that cannot be accomplished by purely cultured microorganisms or improve the metabolic functions of multi-cell systems (Jones and Wang, 2018). Research has shown that *P. putida* can be introduced into microbial consortia as an excellent mcl-PHA producing strain (Huang et al., 2016). At present, artificial microbial consortia are widely used to overcome the shortcomings of the pure culture process to synthesize natural products (Jones et al., 2016; Zhang and Wang, 2016; Liu Y. et al., 2018), and high value-added chemicals (Zhang et al., 2015; Zhang and Stephanopoulos, 2016; Liu X. et al., 2018).

**TABLE 1:** Research on artificial consortia for PHA synthesis.

Strains	Substrates	Type of PHA	Titer (g/L)	Yield^*^ (g/g)	References
*P. putida* KT2440 + *E. coli* MG1655	Glucose + xylose	mcl-PHA	1.32	0.07	This study
*P. putida* KT2440 + *E. coli* MG1655	Glucose + xylose	mcl-PHA	0.54	0.03	Liu et al. (2020)
*P. putida* KT2440 + *E. coli* MG1655	Corn straw hydrolysate	mcl-PHA	0.43	0.02	Liu et al. (2020)
*Ralstonia eutropha* H16 + *Bacillus subtilis* 5119	Sucrose	scl-PHA	2.30	0.08	Bhatia et al. (2018)
*Cupriavidus necator* DSM 428 + *Pseudomonas citronellolis* NRRL B-2504	Apple pulp wastes	P (3HB) and mcl-PHA	1.85	0.11	Rebocho et al. (2020)
*Saccharophagus degradans* 2–40 + *Bacillus cereus*	Xylan	scl-PHA	0.27	N.A.	Sawant et al. (2017)
*Cupriavidus necator* IPT 026 + *Xanthomonas campestris* IBSBF 1867	Palm oil	scl-PHA	6.43	0.05	Rodrigues et al. (2019)
*Aeromonas hydrophila* ATCC7966 + *Acinetobacter junii* BP25	Acetic acid + butyric acid	scl-PHA	2.64	N.A.	Anburajan et al. (2019)
*Synechococcus elongatus cscB + Pseudomonas putida cscAB*	CO_2_	mcl-PHA	0.16	N.A.	Loewe et al. (2017)
*Synechococcus elongatus* PCC 7942*+ Escherichia coli*	CO_2_	scl-PHA	N.A.	N.A.	Hays et al. (2017)
*Synechococcus elongatus* PCC 7942*+ Azotobacter vinelandii* AV3	CO_2_	scl-PHA	N.A.	N.A.	Smith and Francis, (2016)

Values were calculated based on visible data of the original paper with unified to two decimal places; ^*^ the yield coefficient is the ratio of the final PHA, titer to the substrate concentration consumed; N.A., not available.

The synthesis of PHA by a microbial consortium composed of two microorganisms has been extensively studied. The cooperation between the strains allows the possibility to expand the range of available substrates and improves PHA production. Different metabolic functions are carried out in different strains, and they cooperate well with each other to complete the entire task. The interaction of the two strains in the microbial consortium will ultimately affect the production efficiency of the target product. The main challenge facing artificial microbial consortia is the difficulty of maintaining a stable ratio of individually engineered strains in the system during co-culture, and this balance can be disrupted by growth competition and metabolic pressures. We proposed a strategy to design and construct a microbial consortium that synthesizes mcl-PHA from a mixed substrate comprising glucose and xylose based on the “nutrition supply–detoxification” concept (Figure 1). The artificial microbial consortium realized the production of mcl-PHA using “unrelated” carbon sources as substrates without the need for external FFAs. This microbial consortium can efficiently produce mcl-PHA with a maximum titer of 1.32 g/L (Figure 7C), and the yield (0.07 g/g) was increased nearly 2.3 times compared with the previous study (0.03 g/g) (Table 1). In addition, the properties of mcl-PHA can be referred the previous study (Liu et al., 2020), because the monomer compositions of the two are very similar. We further knocked out the *fadD* gene and heterologously expressed an acyl carrier protein thioesterase gene in the *E. coli* mutant strain, which improved the engineered *E. coli* to secrete acetic acid and FFAs. Enhancing the function of *E. coli* is critical for the accumulation of mcl-PHA by *P. putida* in the microbial consortium. Acetic acid and FFAs are used as intermediate metabolic substances between the two strains in the microbial consortium. The ability of *P. putida* to utilize acetic acid and FFAs will directly affect the overall productivity and the conversion efficiency of the initial substrate. Therefore, we weakened the fatty acid β-oxidation and enhanced the acetic acid assimilation pathways of *P. putida*, which increased the production of mcl-PHA and relieved the inhibition of bacterial growth caused by the accumulation of large amounts of acetic acid. However, acetic acid is less important for the intracellular accumulation of mcl-PHA by *P. putida* KT2240 than FFAs, which are the main substrate for PHA accumulation. Rerouting the carbon flux from fatty acid synthesis toward the synthesis of mcl-PHA is a potential strategy to improve the synthesis of mcl-PHA in strains using “unrelated” carbon sources such as acetic acid. In addition, *P. putida* could be further engineered a more ideal PHA production cell factory through various methods, including weakening competing pathways (Wang et al., 2011), strengthening the PHA synthesis pathway (Tobin et al., 2007), modifying the cell size (Liang et al., 2021), and eliminating PHA consumption (Cai et al., 2009). All these approaches can improve the overall production efficiency of mcl-PHA by the microbial consortium.

Competition between the two strains in the microbial consortium for glucose is inevitable. Therefore, we introduced an engineered *E. coli* that preferentially uses xylose. When the microbial consortium uses a composite substrate with a 1:1 mass ratio of glucose and xylose, it can still maintain stable production of mcl-PHA, which shows that the microbial consortium can effectively utilize xylose. This brings us further in the development of microbial consortia to expand the range of substrate utilization. Lignocellulose is the largest biomass resource in nature, and its main components include cellulose, hemicellulose, and lignin (Yang et al., 2007). Following hydrolysis, lignocellulosic biomass mainly yields glucose and a small amount of xylose. Lignocellulose has already been used in the synthesis of PHA (Sandhya et al., 2013; Kucera et al., 2018). The primary method is to convert lignocellulose into fermentable sugars through various ways and then use sugar components to carry out biological fermentation to synthesize PHA (de Souza et al., 2020). However, most studies produced the relatively low-value PHB, and there are few reports on the synthesis of mcl-PHA from lignocellulosic sugars. Researchers have introduced xylose utilization genes into *P. putida* KT2440 to construct an engineered strain that can use xylose to accumulate biomass, but it could not produce mcl-PHA (Le Meur et al., 2012). Here, we were able to use xylose directly through the microbial consortium, and the yield of mcl-PHA has been dramatically improved. Therefore, lignocellulose can be used as a potential substrate for microbial consortia. Using cheap lignocellulosic sugars as a carbon source reduces the production cost compared to fatty acids. Therefore, Using the microbial consortium as a microbial cell factory to produce mcl-PHA offers clear economic advantages.

The biosynthesis results of the co-cultures with different inoculation times have significant differences, indicating that the development of control strategies for fed-batch culture may boost the utilization of mixed sugars to an even higher level, thus maximize the co-culture’s capability of producing mcl-PHA. The two strains in the microbial consortium developed in this study have different substrate preferences, and there is a one-way energy transfer between them. Therefore, when scaling up the microbial consortium to fed-batch, according to the inoculation time verified in the previous period, we consider prioritizing the inoculation of *P. putida* and promoting the accumulation of *P. putida* biomass through the flow of glucose addition mode. Then, at a specific time, xylose was fed, and *E. coli* were inoculated simultaneously to secrete the intermediate metabolites acetic acid and FFAs. The right proportion and feed concentration of mixed sugars to maintain energy metabolism for growth, the secretion of intermediate metabolites, and product formation enable higher productivity of mcl-PHA in co-culture. What needs to be ensured is that the feed concentration should be sufficient to maintain the accumulation of mcl-PHA and not allow it to be decomposed by depolymerase. In addition, prolonging a particular phase of batch culture favors mcl-PHA formation due to the interaction between the two strains. For example, ensuring enough biomass of *P. putida*, optimizing the C/N ratio in the fed-batch stage to promote the secretion of intermediate metabolites may increase the synthesis of mcl-PHA. In short, this requires us to find an equilibrium between maximizing the synthesis of mcl-PHA and the threshold of mixed sugar ratio, feed concentration, and C/N ratio in the fed-batch co-culture phase.

## Conclusion

We optimized a previously developed microbial consortium composed of engineered *E. coli* and *P. putida* to produce mcl-PHA from a mixed substrate of glucose and xylose. We knocked out the *fadD* gene in the genome of *E. coli* Δ4 and expressed an exogenous acyl carrier protein thioesterase gene. The resulting engineered *E. coli* Δ4D (T3) was fermented to produce acetic acid and FFAs with xylose as the sole carbon source, and it exhibited 3.0 and 4.0 fold higher titers than the wild-type bacteria, respectively. *P. putida* KTΔAB (p2-acs-phaJ) has an improved acetic acid assimilation pathway and fatty acid β-oxidation pathway, which enabled the synthesis of mcl-PHA from glucose, acetic acid, and FFAs, while solving the growth inhibition of *E. coli* caused by the accumulation of acetic acid in the culture broth. Finally, we conducted competition control and fermentation optimization for the microbial consortium. The two strains avoided substrate competition and formed a mutually beneficial symbiosis based on the concept of “nutrient supply-detoxification”. The microbial consortium was able to effectively use the mixed substrate of glucose and xylose, the main components of lignocellulosic hydrolysates, to produce mcl-PHA, with a maximum titer of 1.32 g/L. This represents a notable 2.3-fold increase of the mcl-PHA yield compared with previous studies. The further development of the microbial consortium was effective in improving the efficiency of substrate conversion. These results demonstrated that the microbial consortium has excellent potential to produce mcl-PHA from lignocellulosic hydrolysates.

## Data Availability

The original contributions presented in the study are included in the article/Supplementary Material, further inquiries can be directed to the corresponding author.
