# Supplementary material for: Optimization of a Two-Species Microbial Consortium for Improved Mcl-PHA Production From Glucose–Xylose Mixtures
Source: Front Bioeng Biotechnol. 2022 Jan 10;9:794331. doi: 10.3389/fbioe.2021.794331 (PMC8784772; doi:10.3389/fbioe.2021.794331)
Supplement: Supplementary file 1 [file DataSheet1.DOCX]

Supplementary Material

**Table S1.** Strains and plasmids are used in this work.

| Name | Description | Source |
| --- | --- | --- |
| ***Escherichia coli*** |  |  |
| DH5α | F^−^ ϕ80d *lac*ZΔM15Δ(*lac*ZYA-*arg*F)U169 *end*A1 *rec*A1 *hsdR*17 (r_k_^-^, m_k_^+^) *sup*E44λ-*thi*-1 *gyrA*96 *re*lA1 *pho*A | TransGen Biotech |
| S17-1 | *RP4-2(Km::Tn7, Tc::Mu-1) pro-82, LAMpir recA1 thiE1 hsdR17, creC510* | Lab stock |
| MG1655 | *E. coli* K-12 F^−^λ^−^*ilv*G^−^*rfb*-50*rph*-1 | This study |
| ΔD | *E. coli* MG1655, *ΔfadD* | This study |
| Δ4 | *E. coli* MG1655, *ΔptsG, ΔmanZ, ΔatpFH, ΔenvR* | This study |
| Δ4D | *E. coli* MG1655, *ΔptsG, ΔmanZ, ΔatpFH, ΔenvR, ΔfadD* | This study |
| DH5α (T3) | *E. coli* DH5α harboring pET-T3-ACP | This study |
| DH5α (tac) | *E. coli* DH5α harboring pET-tac-ACP | This study |
| ΔD (T3) | *E. coli* ΔD harboring pET-T3-ACP | This study |
| ΔD (tac) | *E. coli* ΔD harboring pET-tac-ACP | This study |
| Δ4D (T3) | *E. coli* Δ4D harboring pET-T3-ACP | This study |
| Δ4D (tac) | *E. coli* Δ4D harboring pET-tac-ACP | This study |
| ***Pseudomonas putida*** |  |  |
| KT2440 | Wild-type | Lab stock |
| KT2440(p2) | *P. putida* KT2440 harboring pBBR1MCS-2 | This study |
| KT2440(p2-*acs*) | *P. putida* KT2440 harboring p2-acs | This study |
| KT2440(p2-*acs-phaJ*) | *P. putida* KT2440 harboring p2-*acs-phaJ* | This study |
| KTΔAB | *P. putida* KT2440, *ΔfadA, ΔfadB* | This study |
| KTΔAB(p2) | *P. putida* KTΔAB harboring pBBR1MCS-2 | This study |
| KTΔAB(p2-*acs*) | *P. putida* KTΔAB harboring p2-acs | This study |
| KTΔAB(p2-*acs-phaJ*) | *P. putida* KTΔAB harboring p2-*acs-phaJ* | This study |
| **Plasmids** |  |  |
| pTKS/CS | p15A replication, Cm^r^, Tet^r^, I-Sce I restriction sites | Lab stock |
| pTKRED | pSC101 replication, temperature-sensitive replication origin, Spc^r^, Para BAD-driven I-Sce I gene, Red recombinase expression plasmid, lac-inducible expression | Lab stock |
| pET28a | The expression vector for *E. coli* | Lab stock |
| pET-T3-ACP | *Ricinus communis* palmitoyl-acyl carrier protein thioesterase inserted into pET28a, T3 promoter | This study |
| pET-tac-ACP | *Ricinus communis* palmitoyl-acyl carrier protein thioesterase inserted into pET28a, tac promoter | This study |
| pBBR1MCS-2 | The expression vector for *P. putida* | Lab stock |
| p2-acs | *acs* inserted into pBBR1MCS-2 | Lab stock |
| p2-*acs-phaJ* | *acs* and *phaJ* inserted into pBBR1MCS-2 | This study |
| pK18mobsacB | Helper knockout plasmid vector | Lab stock |
| pK18-*fadA* | Helper knockout *fadA* vector | This study |
| pK18-*fadB* | Helper knockout *fadB* vector | This study |

**Table S2.** Primers used for the construction of recombinant plasmids.

| Primers^a^ | Sequence (5'→3') |
| --- | --- |
| ***E.coli*** |  |
| fadD up-f | TTGTTTTTAAAGAAAAAGAAACAGC |
| fadD up-r | gtaaatattaccctgttatccctaTTCTTCACCTCTAAAATGCGTG |
| Tet-f | GAACACGCATTTTAGAGGTGAAGAAtagggataacagggtaatatttacg |
| Tet-r | GTCTGACGACTGACTTAACGCTTCTTCACCTCTAAAATGCGTGTTCGTCGTattaccctgttatccctactaagcac |
| fadD down-f | gtgcttagtagggataacagggtaatACGACGAACACGCATTTTAGAGGTGAAGAAGCGTTAAGTCAGTCGTCAGAC |
| fadD down-r | ATCAACCCCAGCTGCGGGTAATAAG |
| RBS-ACP-f | ATACGTATTTAAATCAGGAGTGGAAATGGTTGCCACCGCAG |
| T3-ACP-f_1_ | ccaagcgcgcaattaaccctcactaaagggaacaaaagctgATACGTATTTAAATCAGG |
| T3-ACP-f_2_ | tttcacacaggaaacagctatgaccatgattacgccaagcgcgcaattaac |
| T3-ACP-f_3_ | GAAGATCTtttcacacaggaaacagctatgaccatg |
| ACP-r | CCCTCGAGTTAATGGTGATGATGATGGTGTGCACTTTC |
| tac-ACP-f_1_ | gagcggataacaatttcacacaggaaacagaattcATACGTATTTAAATCAGGAG |
| tac-ACP-f_2_ | ctggcaaatattctgaaatgagctgttgacaattaatcatcggctcgtataatgtgtggaattgtgagcggataacaatttcac |
| tac-ACP-f_3_ | cccgttctggataatgttttttgcgccgacatcataacggttctggcaaatattctgaaatg |
| tac-ACP-f_4_ | GAAGATCTcccgttctggataatgttttttgcgc |
| ***P. putida*** |  |
| *fadA* up-f | CGGGATCCccactcggtggcacgacaac |
| *fadA* up-r | GGAATTCgatggctgttcctcagag |
| *fadA* down-f | GGAATTCgccggtacccaaggccag |
| *fadA* down-r | CCCAAGCTTctgcaacgcaacgaacgcatc |
| *fadB* up-f | GGAATTCaaaaggccaggcaggcc |
| *fadB* up-r | GAAGATCTcaactgatctccacg |
| *fadB* down-f | GAAGATCTgcggtcaacgagctag |
| *fadA* down-r | CCCAAGCTTctccacgccaccgaccacgaacacatcac |
| acs-f | CGggatccTCAAATCTTGGGCAAGGCCGTTG |
| acs-r | CCCAAGCTTATGCCCCAGCCAAGCTACAC |
| phaJ-f | GGAATTCCATATGTCAGCTCGCCACAAAGTTCG |
| phaJ-r | CGggatcctttcacacaggaaacagctatgaccatgattacgc |

^a^Restriction sites are shown in underline.

**Table S3.** Monomer compositions of the mcl-PHA synthesized by the engineered *P. putida*.

| **Strains** | **Monomer compositions (mol%)** | | | | |
| --- | --- | --- | --- | --- | --- |
|  | 3HHX (C6) | 3HO (C8) | 3HD (C10) | 3HDD (C12) | 3HTD (C14) |
| KT2440 | 11.07 ± 3.21 | 52.92 ± 1.13 | 14.89 ± 1.85 | 13.44 ± 2.19 | 7.68 ± 0.97 |
| KT∆AB | 4.64 ± 0.99 | 69.01 ± 3.15 | 13.16 ± 2.79 | 4.42 ± 0.97 | 8.77 ± 1.60 |
| KT2440(p2) | 13.86 ± 1.98 | 58.84 ± 2.21 | 14.87 ± 2.65 | 7.20 ± 0.55 | 5.23 ± 0.59 |
| KT∆AB(p2) | 3.92 ± 1.12 | 64.84 ± 4.17 | 12.57 ± 1.88 | 5.36 ± 1.24 | 13.31 ± 3.16 |
| KT2440(p2-acs) | 11.30 ± 1.36 | 50.65 ± 2.07 | 17.59 ± 3.40 | 10.16 ± 3.19 | 10.30 ± 2.49 |
| KT∆AB(p2-acs) | 2.24 ± 0.46 | 65.06 ± 4.14 | 10.40 ± 1.58 | 9.29 ± 2.88 | 13.01 ± 1.76 |
| KT2440(p2-acs-phaJ) | 9.55 ± 1.50 | 54.02 ± 3.23 | 15.53 ± 3.46 | 9.61 ± 1.40 | 11.29 ± 2.60 |
| KT∆AB(p2-acs-phaJ) | 2.93 ± 1.21 | 68.89 ± 5.16 | 8.38 ± 1.81 | 9.57 ± 1.38 | 10.23 ± 1.22 |

Note: 3HHx: 3-hydroxyhexanoate; 3HO: 3-hydroxyoctanoate; 3HD: 3-hydroxydecanoate; 3HDD: 3-hydroxydodecanoate; 3HTD: 3-hydroxytetradecanoate. Date represent the mean values ± standard deviations (SD) of triplicate measurements from three independent experiments.

**Appendix A Original Gene Sequence of Ricinoyl Carrier Protein Thioesterase**

aaaagaaaagaaaagaaattctcgcgtttgttagctgctttccttccttccttcctctctcgctctccgctggctggctggctgtagaagtatgctggagtaaatttgtgcgacatacagaagattctaaaacctgcttgttcaattttgtgaattccaaaagttgcctatttatcgattgtccaattacatcatggttgctactgcggctgctgctacttcctctttctttccagttccttctcaatctgcggatgctaatttcgataaggcacctgcaagcttaggtggaatcaaattaaaatctacctcttgctctcggggtttacaggttaaggcaaatgcgcaagcccctcccaagataaatggatcctcggtaggattcacaacatctgtggaaactgtgaagaatgacggtgacatgccattaccaccaccccctaggacttttatcaaccaattacctgattggagcatgcttcttgctgctattacaactatctttttggctgctgaaaagcagtggatgatgcttgactggaaaccaaggcggcctgacatgcttatcgacccgtttggtataggtagaattgttcaggatggtcttatttttcgccagaacttctccataagatcatatgaaattggtgctgatcgtacagcatccatagagacattaatgaatcatttacaagaaacggccctcaatcatgttaagactgctggacttcttggggatggatttggttcaaccccagagatgagcaaaaggaacctcatatgggtggttactcggatgcaggttctggtggatcgttacccaacatggggtgatgttgttcaagtagatacttgggtgagtaaatcaggaaagaatggcatgcggcgtgattggtgcgtccgtgatagtagaactggtgaaactttaacgagagcatccagcgtgtgggtgatgatgaataaactgactaggaggttatctaaaattcccgaagaagttcgaggagaaatagagccttattttctgaattctgatcctattgtggatgaggatagcagaaaactgccaaagcttgatgatagcaatgcggactatgtccgcaaaggtctaactcctagatggagtgatctagatatcaaccaacatgttaacaatgtgaaatacattggctggattcttgagagtgctccactgccaatactggagagtcatgaactctctgccattactctggagtataggagggagtgcgggagggacagtgtactgcagtctctgactgctgtatccggtaatggtattggaaatttgggaaatgctggtgatattgagtgccagcacttgcttcgacttgaggatggggctgagatagtgaggggaaggaccgagtggaggccaaagtacagcagcaactttggtattatgggtcagattccagtcgaaagtgcttaagagttgctgtctttgtcgactggagtggcattagtctcctcctaatgtcacaactcacaaagcctttgtagaaatcctccttcgttcagttattggagttatatatgtttcttttttttttttttttgatatatatatatttatatattctttttctcatttcatggaaaaggggaagttgtaagctcgtgtaattaagttgctgaactctttatctctcactttcactctctttctctctcctttgaagtggctctgcttttggaacctgaaagagtcaaatttgtcatgggagaagctctctctctctctctctctctctcgcttgccaacagcctgttcaacttggttggtgttagcatgttccgacatggcccctactcccgattgtctggatgcttcaggattatggcctttattagggggaacacatgtatatttgcggcattcttttattacttttttatcccttgttaatttaggaatcgacagaaaactggatttgatggttcatttattatttttgagtagtagttattatcattga

**Appendix B Ricinoyl carrier protein thioesterase codon-optimized gene sequence**

CATATGTTTCACACAGGAAACAGCTATGACCATGATTACGCCAAGCGCGCAATTAACCCTCACTAAAGGGAACAAAAGCTGATACGTATTTAAATCAGGAGTGGAAAAAAGAAAAGAAAAGAAATTCTCGCGTTTGTTGAGCTGCTTTCCTTCCTTCCTTCCTCTCTCGCTCTCCGCTGGCTGGCTGGCTGTAGAAGTATGCTGGAGTAAATTTGTGCGACATACAGAAGATTCTAAAACCTGCTTGTTCAATTTTGTGAATTCCAAAAGTTGCCTATTTATCGATTGTCCAATTACATCATGGTTGCCACCGCAGCAGCAGCAACCAGCAGCTTTTTTCCGGTTCCGAGCCAGAGCGCAGATGCAAATTTTGATAAAGCACCGGCAAGTCTTGGTGGCATTAAACTGAAAAGCACCAGCTGTAGCCGTGGTCTGCAGGTTAAAGCAAATGCACAGGCACCTCCGAAAATTAACGGTAGCAGCGTTGGTTTTACCACCAGTGTTGAAACCGTTAAAAATGATGGTGATATGCCGCTGCCTCCGCCTCCGCGTACCTTTATTAACCAGCTGCCGGATTGGAGCATGCTGCTGGCAGCAATTACCACCATTTTTCTGGCAGCAGAAAAACAGTGGATGATGCTGGATTGGAAACCGCGTCGTCCGGATATGCTGATTGATCCGTTTGGTATTGGTCGTATTGTTCAGGATGGTCTGATTTTTCGTCAGAACTTTAGCATTCGCAGCTATGAAATTGGTGCAGATCGTACCGCAAGCATTGAAACCCTGATGAATCATCTGCAAGAAACCGCACTGAATCATGTTAAAACCGCAGGTCTGTTAGGTGATGGTTTTGGTAGCACACCGGAAATGAGCAAACGTAATCTGATTTGGGTTGTTACCCGTATGCAGGTTCTGGTTGATCGTTATCCGACCTGGGGTGATGTTGTTCAGGTTGATACCTGGGTTAGCAAAAGCGGTAAAAATGGTATGCGTCGTGATTGGTGTGTTCGTGATAGCCGTACCGGTGAAACACTGACCCGTGCAAGCAGCGTTTGGGTTATGATGAATAAACTGACACGTCGCCTGAGCAAAATTCCGGAAGAGGTTCGTGGTGAAATTGAACCGTATTTTCTGAATAGCGATCCGATCGTTGATGAAGATAGTCGTAAACTGCCGAAACTGGATGATAGCAATGCAGATTATGTTCGCAAAGGTCTGACACCGCGTTGGAGCGATCTGGATATCAACCAGCATGTTAATAACGTGAAATATATCGGCTGGATTCTGGAAAGCGCACCGCTGCCGATCCTGGAAAGCCATGAACTGAGCGCGATTACCCTGGAATATCGTCGTGAATGTGGTCGTGATTCAGTTCTGCAGAGCCTGACCGCAGTTAGCGGTAATGGTATTGGCAATCTGGGTAATGCCGGTGATATTGAATGTCAGCATCTGCTGCGTCTGGAAGATGGTGCAGAAATTGTTCGTGGTCGTACCGAATGGCGTCCGAAATATAGCAGCAATTTTGGTATTATGGGTCAGATTCCGGTTGAAAGTGCACACCATCATCATCACCATTAAGAGTTGCTGTCTTTGTCGACTGGAGTGGCATTAGTCTCCTCCTAATGTCACAACTCACAAAGCCTTTGTAGAAATCCTCCTTCGTTCAGTTATTGGAGTTATATATGTTTCTTTTTTTTTTTTTTTTGATATATATATATTTATATATTCTTTTTCTCATTTCATGGAAAAGGGGAAGTTGTAAGCTCGTGTAATTAAGTTGCTGAACTCTTTATCTCTCACTTTCACTCTCTTTCTCTCTCCTTTGAAGTGGCTCTGCTTTTGGAACCTGAAAGAGTCAAATTTGTCATGGGAGAAGCTCTCTCTCTCTCTCTCTCTCTCTCGCTTGCCAACAGCCTGTTCAACTTGGTTGGTGTTAGCATGTTCCGACATGGCCCCTACTCCCGATTGTCTGGATGCTTCAGGATTATGGCCTTTATTAGGGGGAACACATGTATATTTGCGGCATTCTTTTATTACTTTTTTATCCCTTGTTAATTTAGGAATCGACAGAAAACTGGATTTGATGGTTCATTTATTATTTTTGAGTAGTAGTTATTATCATTGAGCGGCCGC


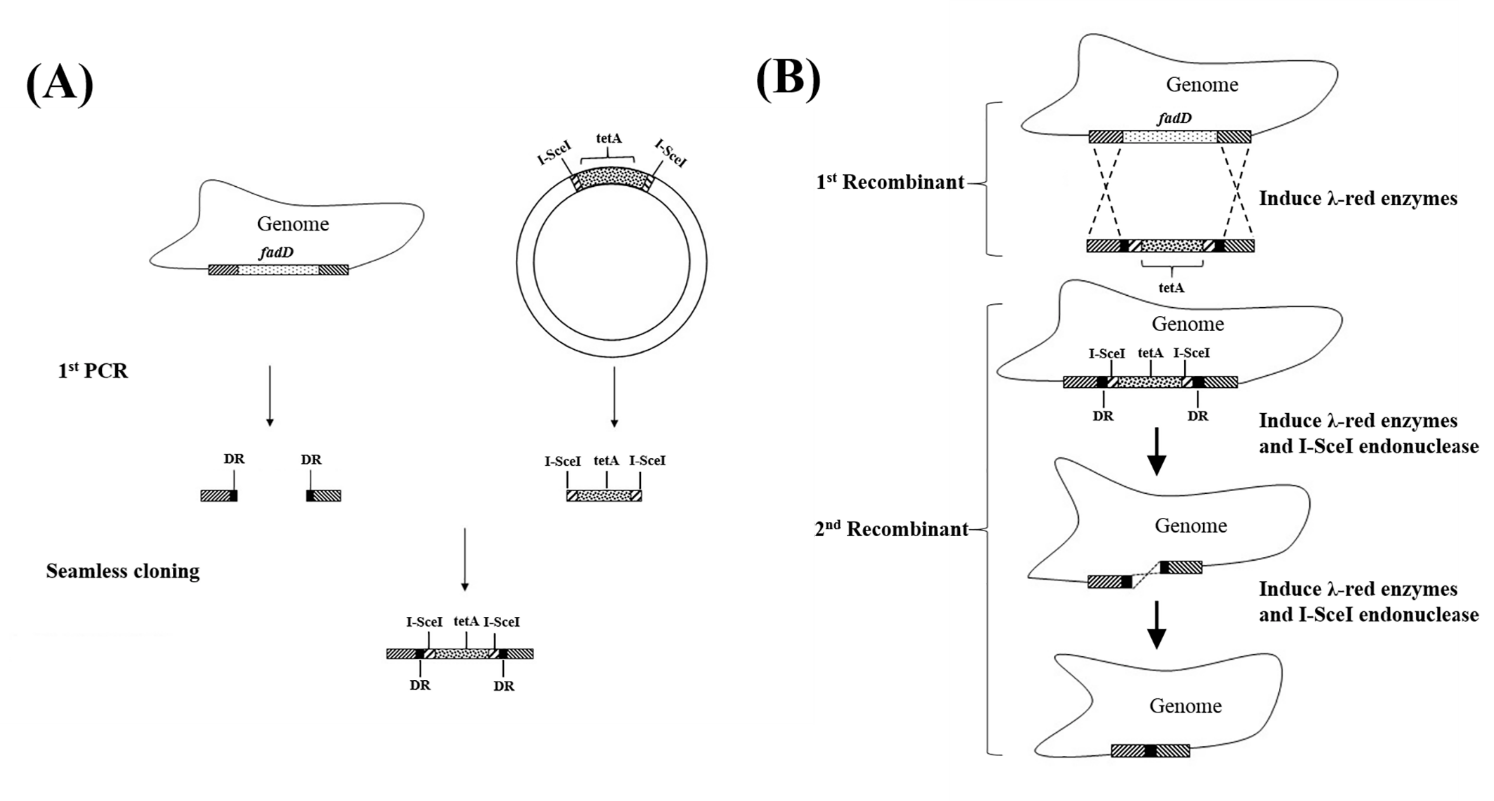


**Supplementary Figure 1.** Strategies for scarless chromosomal gene deletion. (A) Genome editing cassettes are constructed by first round of PCR and seamless cloning. (B) Recombinants are obtained by first round of recombineering, and the TetA marker were released by simultaneous induction of I-SceI and Red recombinase expression. DR for duplicate region; I-sceI, I-SceI endonuclease recognition site.


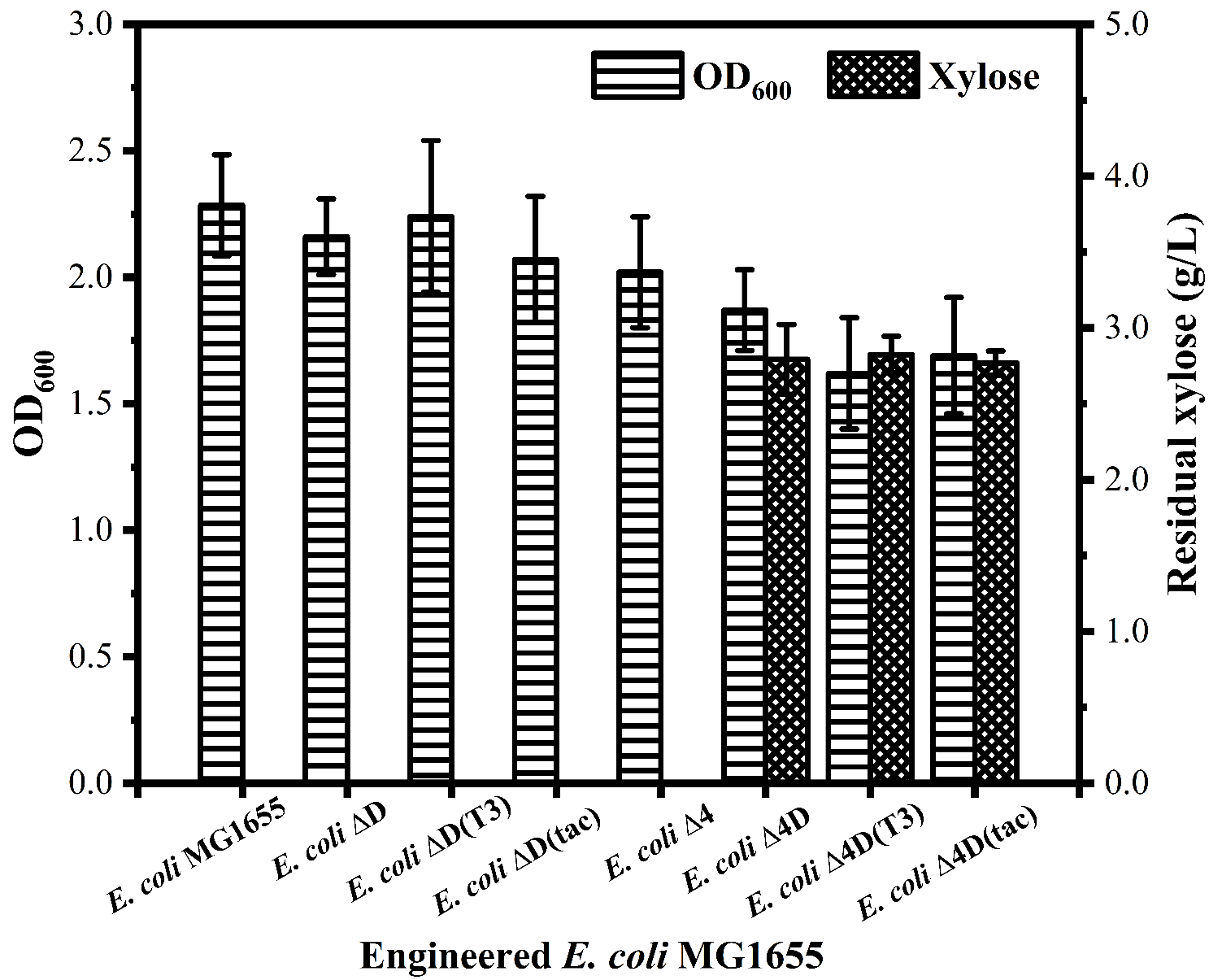
**Supplementary Figure 2.** The OD_600_ and residual xylose at the end of fermentation of engineered *E. coli* with acetic acid as the sole substrate.

**
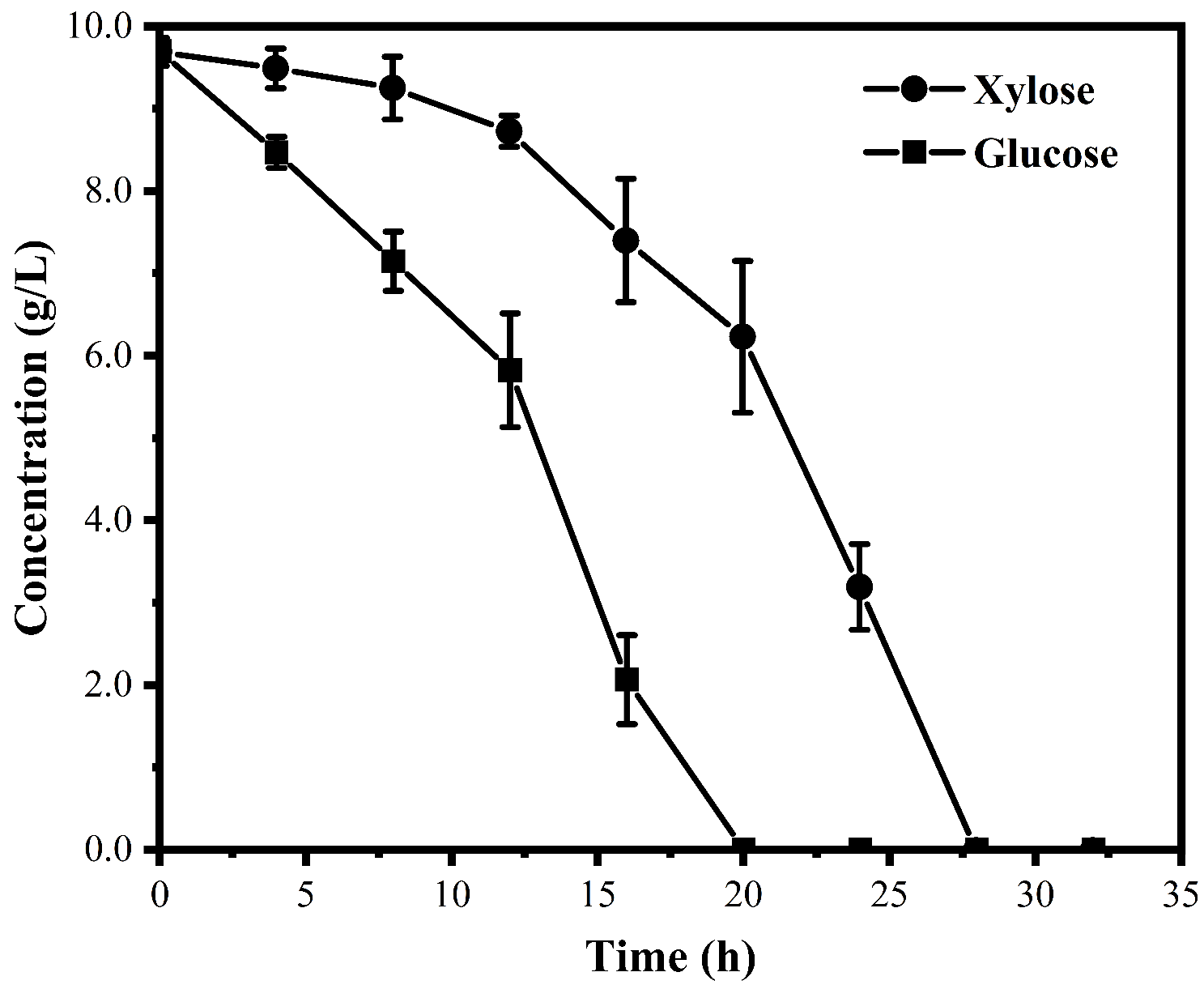
Supplementary Figure 3.** Consumption of glucose and xylose during the fermentation process of the artificial microbial consortium when the inoculation ratio of the two bacteria is *E. coli*: *P. putida*=1:2.
